# Harmonized Sequential Extraction Parameters for Soil Organic Matter Fractions Across Contrasting Land-Use Soils: Optimization and Mass-Balance Assessment

**DOI:** 10.3390/molecules31213880

**Published:** 2026-10-27

**Authors:** Yunchuan Dai, Yi Sun, Qiqi Zhang, Ning An, Sibiao Zhao, Deren Miao

**Affiliations:** 1 School of Chemistry and Chemical Engineering, Kunming University, Kunming 650214, China; 2 Kunming Institute for Food and Drug Control, Kunming 650118, China

**Keywords:** humic substances, sequential fractionation, humin, mass-balance recovery, organo-mineral association

## Abstract

Fine fractionation of soil organic matter (SOM) underpins the interpretation of element speciation in different SOM pools, yet sequential extraction methods are mostly optimized for single soil types, limiting inter-study comparability. We optimized extraction time and concentration for six SOM fractions—exchangeable SOM (Ex), humic acid (HA), fulvic acid (FA), iron-bound humin (HMi), clay-bound humin (HMc), and insoluble humin (HMr)—using forest, vegetable, and farmland soils from Lincang, Yunnan, and characterized the extraction residues by FTIR, SEM, and EEM analyses. Optimal parameters fell within 8–12 h and 0.5–1.0 mol L^−1^: Ex, 1 mol L^−1^ NH_4_Ac (pH 5.0, 12 h); HA/FA, 1 mol L^−1^ NaOH + 0.05 mol L^−1^ Na_4_P_2_O_7_ (12 h); HMi, 0.5 mol L^−1^ H_2_SO_4_ (8 h), then 0.5 mol L^−1^ NaOH + saturated Na_2_SO_4_ (12 h); HMc, 1 mol L^−1^ H_2_SO_4_–HF (1:1, *v*/*v*, 12 h), then the same alkali extraction. Total SOM extraction rates reached 90.05% ± 1.12% to 96.97% ± 1.27% (*n* = 3); residues showed progressive stripping from weakly to strongly bound fractions, and fraction distributions differed consistently among land-use types. This framework provides a methodological basis for cross-soil SOM fractionation.

## 1. Introduction

Soil organic matter (SOM) holds the bulk of the carbon stored in soils, and its content and composition shape the mobility, retention, and bioavailability of nutrients and potentially toxic elements [1,2]. Its carboxyl and phenolic hydroxyl groups complex and chelate metal ions and mediate redox reactions; through this chemistry, SOM controls how elements partition between the solid and solution phases and how readily they move with runoff or leaching water. SOM likewise carries nitrogen, phosphorus, sulfur, and many trace elements through their biogeochemical cycles [3]. Through ligand exchange and co-precipitation with Fe and Al oxides and clay minerals, it forms organo-mineral complexes that retain elements in the soil profile over long periods [4,5]. Understanding how elements migrate, transform, and are retained in soil thus calls for separating SOM into finer, compositionally distinct fractions.

Chemically, SOM is heterogeneous: a continuum of components that differ in origin, degree of humification, molecular weight, and mode of association with minerals [6]. Humic substances, which constitute the bulk of SOM, fall into humic acid (HA), fulvic acid (FA), and humin (HM) according to their solubility in alkali and acid, and the three fractions interact with elements in distinct ways. FA is low in molecular weight and rich in functional groups; it stays soluble across a wide pH range, forms mobile complexes with metals, and promotes their leaching and redistribution. HA, larger and more aromatic, binds metal ions with greater capacity and affinity and precipitates under acidic conditions [7]. HM is what remains after alkali and acid extraction; bound tightly to soil minerals, it holds elements in a comparatively persistent form. The alkali dissolution–acid precipitation method and the standardized extraction procedures built on this scheme [8,9] have long provided the methodological link between SOM composition and the forms in which elements occur in soil. HM itself was long regarded as a single, inert, stable carbon pool, and its contribution to element fixation and soil functioning was underestimated [10]. Its internal heterogeneity drew little attention until Pallo [11] subdivided HM. Dou et al. [12] went further, partitioning HM into Fe-bound humin (HMi), clay-bound humin (HMc), and insoluble humin (HMr), and reporting that the three differ in content and distribution. These fractions owe their existence to the reactive surfaces of Fe oxides and clay minerals, on which soil organic matter sorbs and is stabilized [4], and the same associations shape how the fractions interact with metal ions: humin takes up Cu(II) as inner-sphere complexes on its surface carboxyl and hydroxyl groups [13]; in paddy soil, HA sequesters germanium (Ge) through stable complexation, while FA forms soluble complexes that act as Ge carriers [14]; and the fractions differ in their capacity to reduce Cr(VI) [15]. Any attempt to attribute the migration or fixation of an element to a particular organic matter fraction first requires that the fractions be cleanly separated.

In sequential chemical extraction, successive reagents strip the fractions stepwise [16]. Representative schemes include the alkali dissolution–acid precipitation method and its modifications [8], the standardized extraction procedure of the International Humic Substances Society (IHSS) [9], and the two-step procedure of acid pretreatment followed by alkali extraction for humin subfractions [11,12]. Yet sequential extraction remains operationally defined: what a fraction contains depends on the reagent, its concentration, and the reaction time [17], and the key parameters are set largely by experience, mostly for individual soils or single soil types. Parameter choice affects more than the amount recovered: the spectroscopic features and molecular composition of the extract shift [18], fractions cross-contaminate each other [19], and the correspondence between the fractions obtained and functional carbon pools blurs [20]. Where optimization has been attempted, it has usually targeted a single step or a single soil type; Weber et al. [21], for instance, adjusted the conditions for isolating humin from mineral soils. Land-use differences aggravate the problem. Forest soils receive mainly litter inputs and are more highly humified [22]; farmland and vegetable soils take in more exogenous organic matter under tillage and fertilization, with a larger share of labile fractions [23,24]; and land-use change further alters the sources of organic matter inputs and the structure of aggregates [25,26]. Confronted with different soils, researchers typically adjust the extraction parameters for each, so the fractions obtained may share a name without sharing the same chemistry. Differences generated in this way may be mistaken for land-use effects, and conclusions on element behavior drawn from a single soil cannot be compared across soils on a common scale. The current methodological system thus has three interrelated defects: extraction parameters are set empirically and vary from soil to soil; extraction completeness is rarely examined at the level of mass balance; and the chemical rationale of the extraction sequence lacks evidence at the residue level [19]. If the amount of a fraction extracted reaches a plateau once the reagent is strong enough to break the dominant bonds holding it, and if the plateau regions of different soils overlap, extraction parameters in principle need not vary with soil type. We therefore hypothesize that fixing the reagent concentration and extraction time of every fraction within this shared plateau region would remove the “same name, different substance” problem, so that a single parameter system could make the mass balance and yield fractions comparable across soils. Two schemes are the most widely used. The IHSS standard procedure [9] is fully standardized but restricted to alkali-soluble HA and FA. The humin fractionation scheme of Pallo [11], as adapted by Dou et al. [12], extends fractionation to humin subfractions (HMi, HMc, HMr) but leaves reagent concentrations and reaction times to empirical, soil-specific choice. Section 2.3 compares the strengths and limitations of these schemes with the present framework.

Forest, farmland, and vegetable soils in Lincang, Yunnan Province, were selected as test materials. We optimized the reagent concentration and extraction time for each fraction through single-factor and two-factor full-factorial experiments. The residues left after each extraction step were characterized by Fourier-transform infrared (FTIR) spectroscopy, scanning electron microscopy (SEM), and three-dimensional excitation–emission matrix (EEM) fluorescence spectroscopy, and these characterizations guided the final choice of a harmonized parameter set for all SOM forms. We then verified extraction completeness under the harmonized conditions by mass balance, using the SOM extraction rate as the indicator. For comparison, we set this system against the classical schemes, which fix the extraction sequence but leave its parameters to empirical choice [11,12], and against optimizations that target one step only [21]. Our goal was to develop a harmonized parameter system for sequential extraction that makes SOM fractionation data from different soils, and the mechanisms of element migration and retention inferred from them, comparable across soils.

## 2. Results and Discussion

### 2.1. Optimization of Sequential Extraction Conditions for Soil Organic Matter Fractions

#### 2.1.1. Single-Factor Experiments

Ex is not a humus fraction per se but the weakly bound, free organic matter associated with soil colloids. It was extracted first so that exchangeable substances would not interfere with the subsequent separation of HA/FA and humin, keeping the operational boundaries between fractions clear.

(1) Exchangeable fraction (Ex)

**Figure 1:**
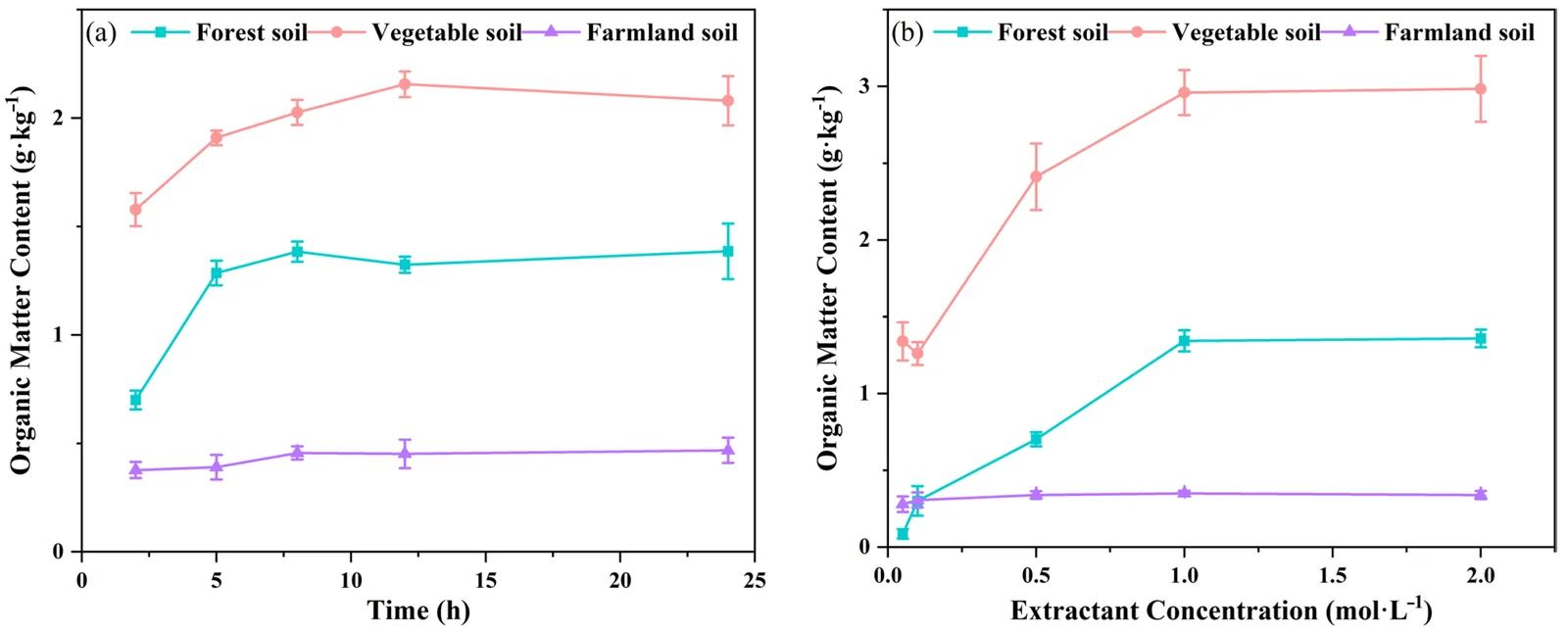
Optimization of Ex extraction: (**a**) extraction time; (**b**) extractant concentration (*n* = 3).

Single-factor results (Figure 1) showed that Ex extraction yields initially increased and then reached a plateau as extraction time and NH_4_Ac concentration increased, stabilizing within 0.5–1.0 mol L^−1^ and 8–12 h. Ex is a weakly bound fraction whose release follows an ion-exchange equilibrium: once the extractant is strong enough to displace free organic matter from soil colloid surfaces, further intensification dissolves more inorganic salt without improving organic carbon recovery. Based on the optimal plateau regions common to all three soils, the unified extraction condition for Ex was set at 1 mol L^−1^ NH_4_Ac (pH 5.0) for 12 h.

(2) Humic acid (HA) and fulvic acid (FA)

**Figure 2:**
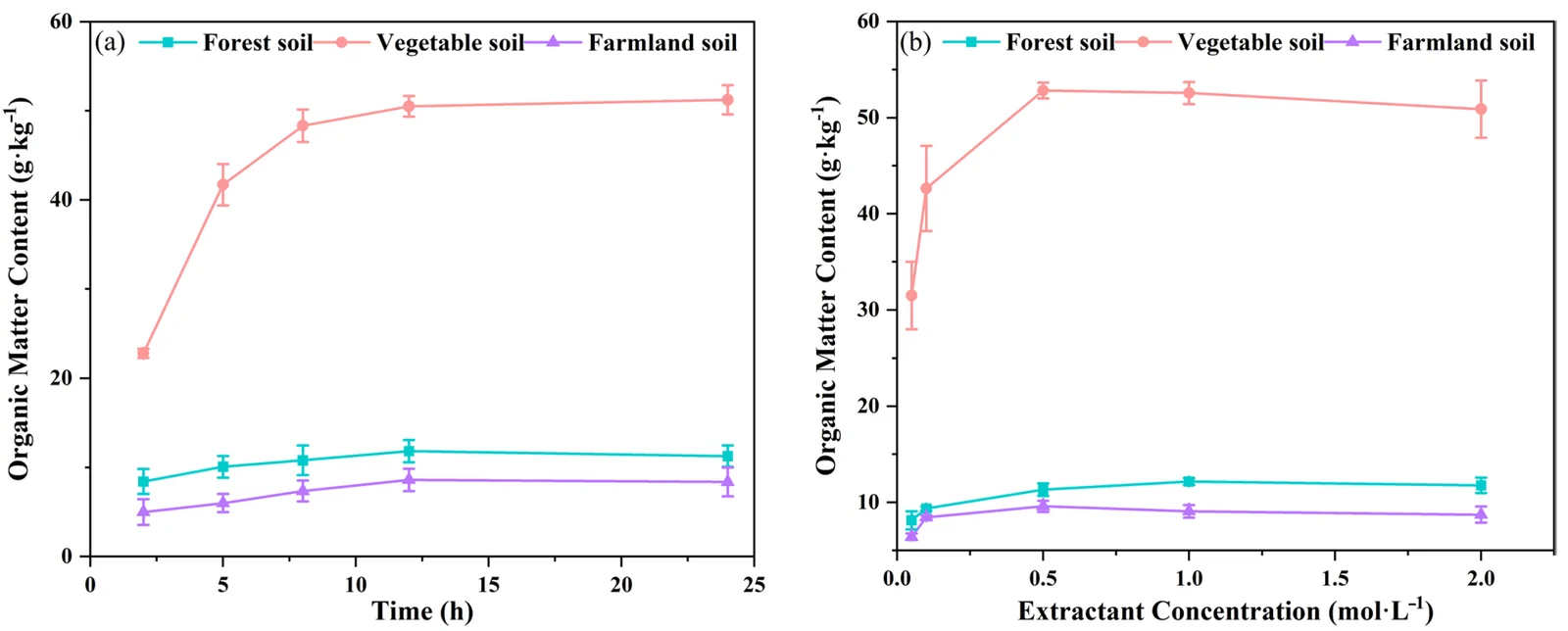
Optimization of HA extraction: (**a**) extraction time; (**b**) extractant concentration (*n* = 3).

**Figure 3:**
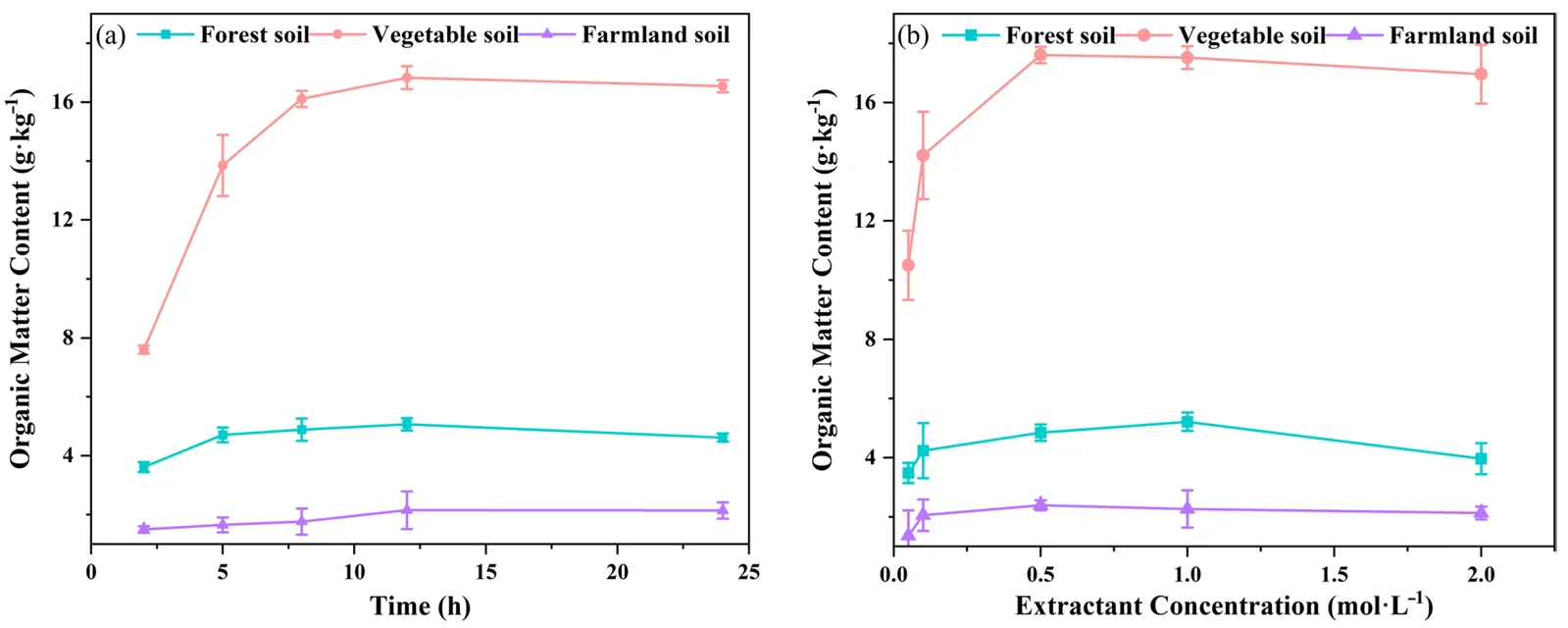
Optimization of FA extraction: (**a**) extraction time; (**b**) extractant concentration (*n* = 3).

The extraction of HA and FA employed a NaOH–Na_4_P_2_O_7_ mixed system, which disrupts organo-mineral complexes through the combined action of alkaline dissolution and chelation, thereby releasing the humic fractions. Single-factor results (Figure 2 and Figure 3) showed that the extraction trends for HA and FA were highly consistent: extraction yields increased with rising NaOH concentration and prolonged extraction time, entering a plateau within the 0.5–1.0 mol L^−1^ and 8–12 h range.

The release of HA and FA is primarily controlled by pH-driven deprotonation of functional groups; once the alkalinity is sufficient to fully deprotonate carboxyl and phenolic hydroxyl groups, humic molecules desorb and dissolve from mineral surfaces [27]. Further increases in alkali concentration may, however, induce organic matter condensation or excessive dissolution of silicates, leading to diminishing marginal returns in extraction efficiency. Based on the optimal plateau regions common to all three soils, the unified extraction condition for HA/FA was set at 1 mol L^−1^ NaOH + 0.05 mol L^−1^ Na_4_P_2_O_7_ for 12 h.

(3) Iron-bound humin (HMi)

**Figure 4:**
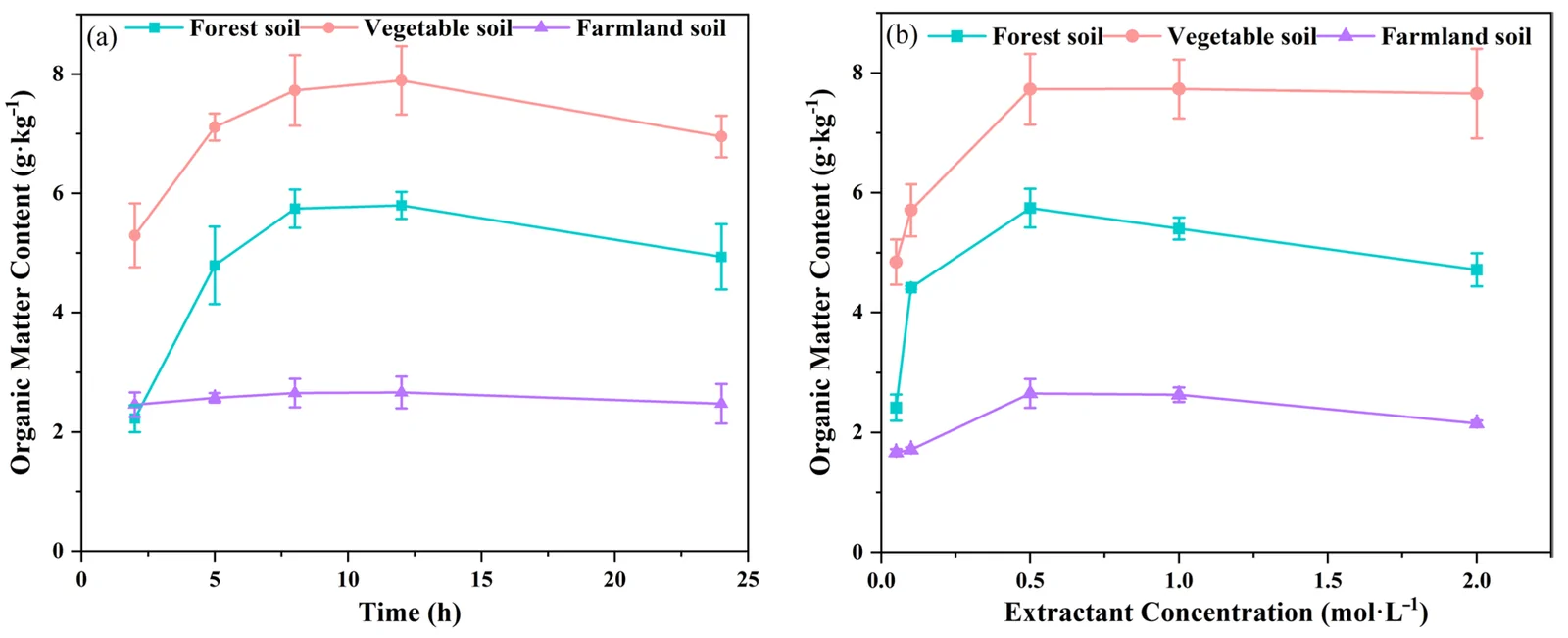
Optimization of HMi-1 extraction: (**a**) extraction time; (**b**) extractant concentration (*n* = 3).

The extraction of HMi employed an “acid destruction–alkaline extraction” two-step method. The method relies on the selective dissolution of iron oxides using dilute sulfuric acid, thereby releasing organic matter bridged by Fe, followed by alkaline extraction of the liberated HMi fraction. Single-factor results (Figure 4) for HMi-1 (acid extraction step) showed that the three soil types responded consistently to H_2_SO_4_ concentration and reaction time, both entering a plateau at 0.5 mol L^−1^ and 8 h. The high-value region was concentrated at a moderate extraction time (8 h) and moderate concentration (0.5 mol L^−1^), indicating that iron oxide dissolution can be efficiently achieved under mild acidic conditions. Prolonging time or increasing acidity beyond this range did not improve HMi-1 release. It could damage organic matter structures or dissolve other mineral phases.

**Figure 5:**
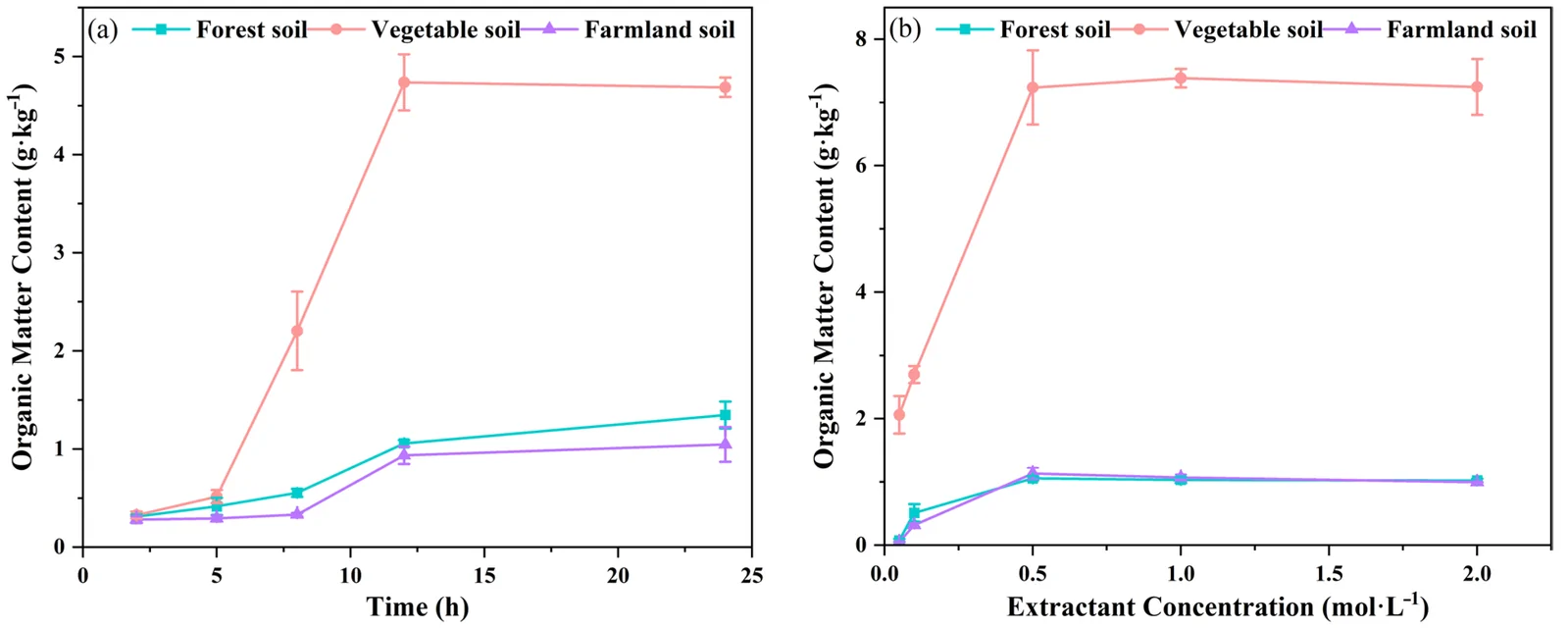
Optimization of HMi-2 extraction: (**a**) extraction time; (**b**) extractant concentration (*n* = 3).

For HMi-2 (alkaline extraction step), the optimization results (Figure 5) showed that high values were concentrated at 12 h and 0.5–1.0 mol L^−1^. Forest and vegetable soils achieved higher extraction yields at elevated alkali concentrations. Farmland soil was more stable at lower alkali concentrations. This reflects differences in the kinetics of organic matter transfer to the liquid phase after acid treatment. These differences arise from variations in clay and mineral composition. Integrating extraction completeness and operational consistency across the three soil types, the unified conditions were set as HMi-1: 8 h, 0.5 mol L^−1^ H_2_SO_4_; HMi-2: 12 h, 0.5 mol L^−1^ NaOH + saturated Na_2_SO_4_.

(4) Clay-bound humin (HMc)

**Figure 6:**
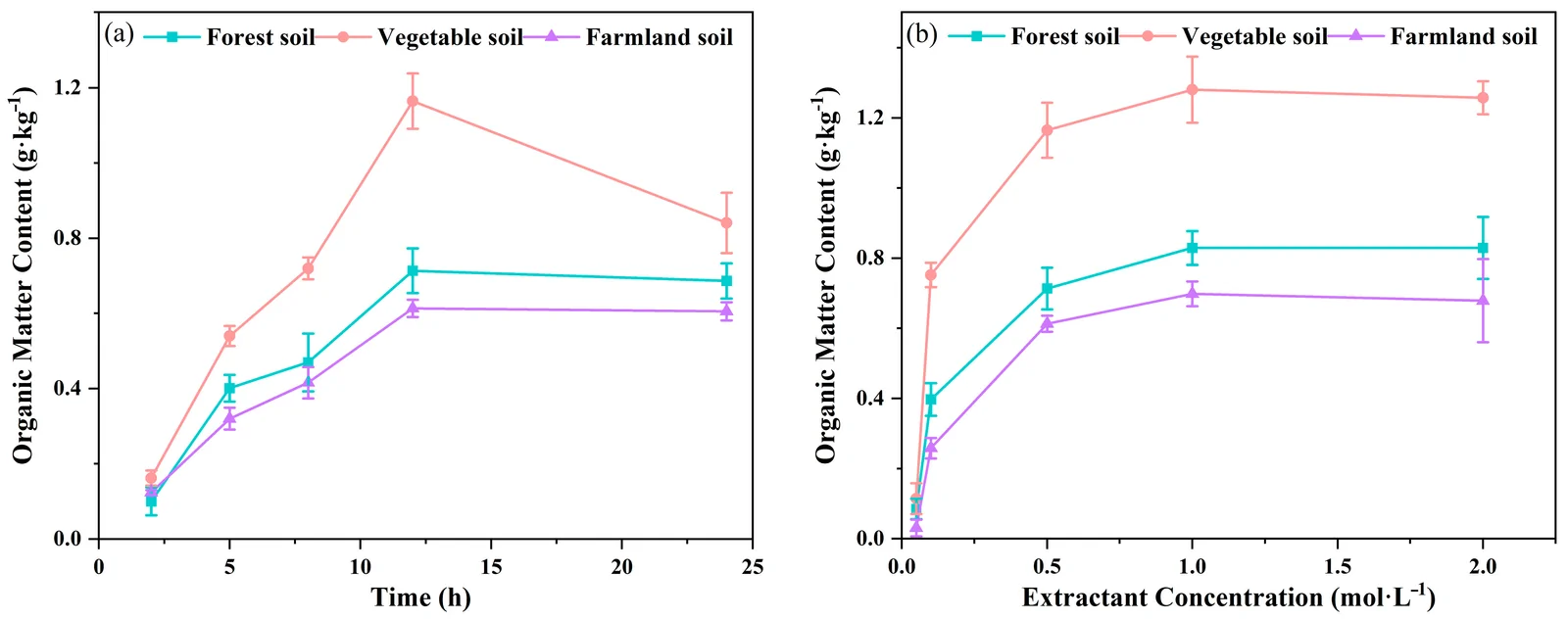
Optimization of HMc-1 extraction: (**a**) extraction time; (**b**) extractant concentration (*n* = 3).

The extraction of HMc also employed a two-step method: the first step used H_2_SO_4_–HF to disrupt silicate mineral lattices, releasing organic matter physically entrapped or chemically bonded within clay minerals; the second step used an alkaline solution to extract the liberated HMc. Single-factor results (Figure 6) for HMc-1 (acid extraction step) showed that the overall extraction yield of this fraction was relatively low and the curves were flat, reflecting the high difficulty of releasing clay-bound organic matter. All three soil types achieved higher efficiency within the 1 mol L^−1^ and 8–12 h range. The high-value region was near 8–12 h and 1.0 mol L^−1^. The introduction of HF significantly enhanced the dissolution efficiency of silicate minerals. However, the lattice stability of clay minerals sets a kinetic barrier that limits further release of organic matter.

**Figure 7:**
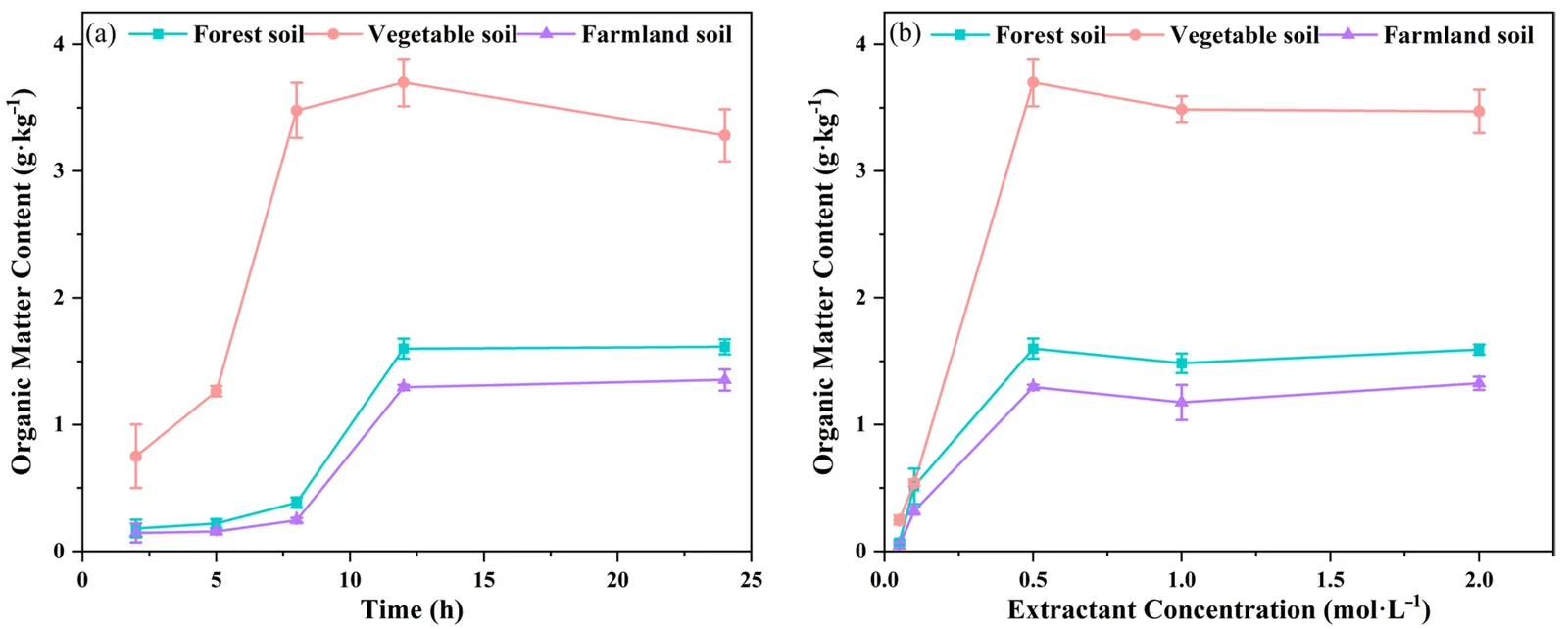
Optimization of HMc-2 extraction: (**a**) extraction time; (**b**) extractant concentration (*n* = 3).

For HMc-2 (alkaline extraction step), single-factor results (Figure 7) showed that high values for all three soil types were concentrated at 8–12 h and 0.5 mol L^−1^, and further increases in concentration or time did not yield significant gains. This suggests that the organic matter exposed after acid treatment can be effectively dissolved under mild alkaline conditions; excessive intensification of extraction conditions may instead induce organic matter reprecipitation or mineral impurity interference. Integrating extraction efficiency and reagent stability, the unified extraction conditions were determined as HMc-1: 12 h, 1 mol L^−1^ H_2_SO_4_ + HF; HMc-2: 12 h, 0.5 mol L^−1^ NaOH + saturated Na_2_SO_4_.

#### 2.1.2. Comprehensive Experiments and Range Analysis

According to the range (*R*) values derived from the comprehensive experiments (Table 1 and Table 2), the extractant concentration exhibited the largest range for Ex extraction across all three soil types, identifying it as the key factor influencing Ex content. Based on the *k* values at each level, the unified extraction condition for Ex was preliminarily determined as 1 mol L^−1^ NH_4_Ac (pH 5.0) for 12 h. During HA and FA extraction, extraction time showed the largest range for forest and farmland soils, whereas extractant concentration showed the largest range for vegetable soil. The unified extraction condition for HA/FA was determined as 1 mol L^−1^ NaOH + 0.05 mol L^−1^ Na_4_P_2_O_7_ for 12 h. For HMi and HMc, both acid and alkaline extraction steps showed the largest range for extractant concentration, identifying it as the key factor influencing their contents. The unified conditions were determined as HMi-1: 8 h, 0.5 mol L^−1^ H_2_SO_4_; HMi-2: 12 h, 0.5 mol L^−1^ NaOH + saturated Na_2_SO_4_; HMc-1: 12 h, 1 mol L^−1^ H_2_SO_4_ + HF; HMc-2: 12 h, 0.5 mol L^−1^ NaOH + saturated Na_2_SO_4_.

To further evaluate the statistical significance of main effects, analysis of variance (ANOVA) was performed on the comprehensive experimental results for each soil organic matter fraction. Using Ex extraction across the three soil types as an illustrative example (Table 3), the F-values for extraction time were uniformly lower than those for extractant concentration (forest soil: 9.08 and 90.3; vegetable soil: 23.1 and 127; farmland soil: 26.7 and 46.2, respectively), yielding a factor hierarchy consistent with that obtained from range analysis. For the remaining SOM fractions, the factor hierarchy determined by ANOVA was largely in agreement with range analysis, with the sole exception of HA and FA in farmland soil, where ANOVA identified extractant concentration as the dominant factor—a discrepancy with the range analysis outcome. The complete range analysis tables and ANOVA tables for HA, FA, HMi, and HMc across all three soil types are provided in Supplementary Materials (Tables S1–S58).

#### 2.1.3. Effects of Extraction Time and Extractant Concentration on SOM Fractions

Single-factor and comprehensive experiments showed that SOM fraction release did not increase linearly with extraction time or extractant concentration. Instead, it exhibited a characteristic “plateau effect.” Prolonged extraction time or elevated concentration beyond the optimal range did not produce significant additional gains and could instead induce organic matter condensation, excessive mineral dissolution, or structural damage [21,28]. The mechanism is plain: once the extractant is strong enough to rupture the relevant bonds, the target fraction is largely released, and what remains is protected by stronger mineral associations or locked in more condensed structures beyond the reach of the reagent [4,29].

The differential responses of individual SOM fractions to extraction conditions track their binding mechanisms. Ex release follows ion-exchange equilibrium, and the largest range for concentration in the comprehensive experiments indicates that NH_4_Ac concentration directly determines the displacement efficiency of free organic matter on colloid surfaces. HA/FA release is controlled by alkalinity-driven deprotonation of functional groups. Forest and farmland soils showed the largest range for time. Vegetable soil showed the largest range for concentration. This reflects differences in alkaline-dissolution kinetics under varying total organic matter backgrounds [27]. Both HMi and HMc used the “acid destruction–alkaline extraction” two-step approach. In comprehensive experiments, acid/alkali concentration showed the largest range. This indicates that the dissolution efficiency of iron oxides and silicate minerals is the critical step limiting organic matter release. The kinetics are controlled by mineral lattice stability, not by simple time extension [10].

#### 2.1.4. Structural Evolution of Fractionated Residues and Validation of Extraction Sequence Rationality

We validated the chemical rationality of the extraction sequence by characterizing the original soil (F1) and stepwise extraction residues (F2–F4) with FTIR, SEM, and EEM. F2 denotes residue after removal of Ex and HA/FA; F3 after removal of HMi; and F4 after removal of HMc.

(1) FTIR spectroscopy: Gradient evolution of functional group composition

**Figure 8:**
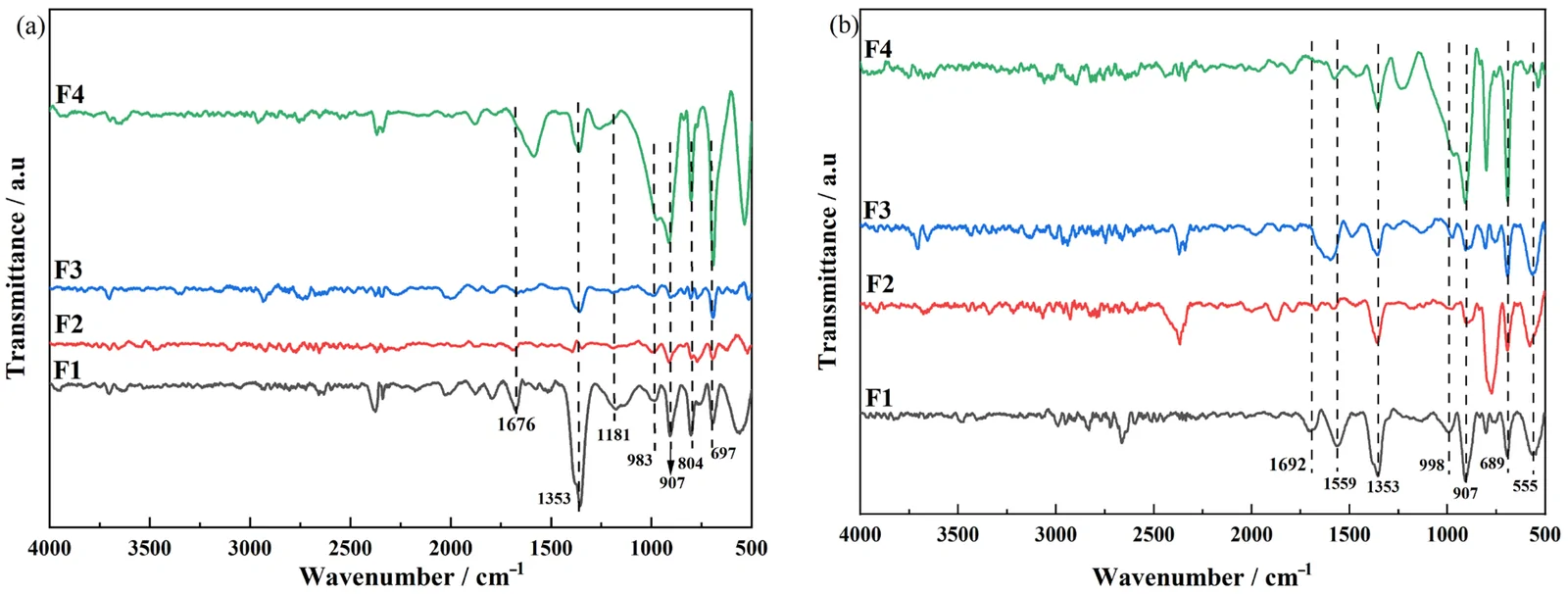
FTIR spectra of soils after fractional extraction: (**a**) forest soil; (**b**) vegetable soil. F1, original soil; F2–F4, residues after removal of Ex and HA/FA, HMi, and HMc, respectively. The farmland soil spectra are provided in Figure S1.

FTIR spectra (Figure 8) showed that, with increasing extraction depth, the organic-matter characteristic bands attenuated stepwise, whereas the mineral-skeleton bands became progressively dominant. F1 displayed clear absorption at 1676–1692 cm^−1^ (C=O stretching of carboxyl/amide groups), 1559–1589 cm^−1^ (aromatic C=C skeletal vibration and asymmetric COO– stretching of carboxylate groups), 1353–1356 cm^−1^ (C–O stretching/O–H bending), and 1181 cm^−1^ (C–O stretching of polysaccharide-like components overlapping the Si–O envelope of silicates) [30], indicating that the original soils were enriched in oxygen-containing functional groups and aromatic structures, with organic matter as a dominant component. In F2, these bands were markedly weakened, demonstrating effective removal of the alkali-soluble humic fractions; by F3, they were further reduced, reflecting the release of iron-bound organic matter. F4 was dominated by mineral-skeleton absorption at 983–998 cm^−1^ (Si–O asymmetric stretching), 907–914 cm^−1^ (Al–O–H bending of layer silicates), 777–804 cm^−1^ (Si–O symmetric stretching of quartz), and 555–697 cm^−1^ (Al–O–Si or metal–oxygen bending) [31], with inorganic components contributing dominantly. The weak residual dip near 1676 cm^−1^ in F4 is assigned to H–O–H bending of adsorbed water rather than to residual organic C=O.

This gradient change is consistent with the intended extraction sequence and the binding-strength gradient at the molecular vibration level. Weakly bound Ex and alkali-soluble HA/FA were removed first, iron oxide-bound HMi was released next, and silicate-bound HMc was disrupted last, a chemical stripping order from weak to strong [32]. This enhancement partly comes from progressive organic matter removal rather than new mineral associations; nor can FTIR exclude cross-contamination on its own. It is used here as corroborating, not decisive, evidence. These residue-level signals also do not establish chemical equivalence between the same fraction obtained from different soils.

(2) SEM: Co-evolution of micro-morphology

**Figure 9:**
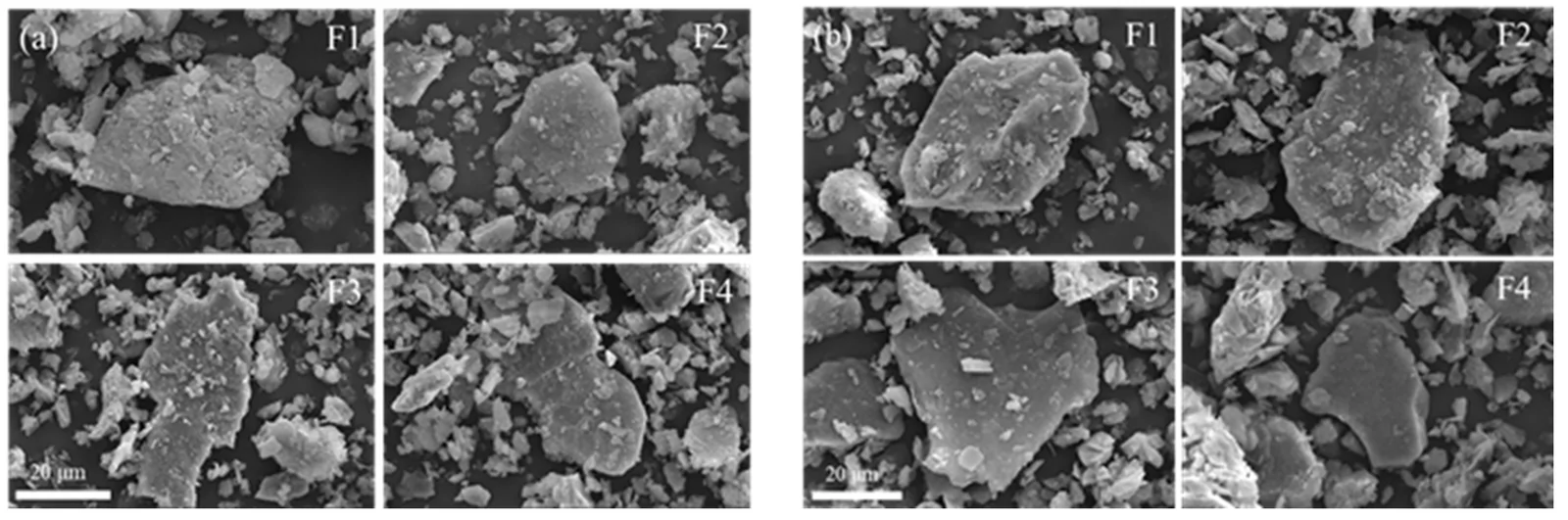
SEM micrographs of soils after sequential extraction: (**a**) forest soil; (**b**) vegetable soil. F1, original soil; F2–F4, residues after removal of Ex and HA/FA, HMi, and HMc, respectively. The farmland soil micrographs are provided in Figure S2.

SEM characterization (Figure 9) revealed that the original soil F1 was covered by a flocculent organic layer. With progressive extraction, the organic membrane gradually peeled off; by the F3–F4 stages, dense mineral skeletons were fully exposed. The same sequence appeared in all three soils: the organic coating disappeared first, the mineral skeleton was then exposed, and the residue surface finally densified. The reproducibility of this sequence across the three land-use soils supports the general applicability of the stripping approach beyond any single soil type. These microscopic observations are consistent with the FTIR results.

(3) Three-dimensional fluorescence spectroscopy: Stepwise depletion of organic matter active components

**Figure 10:**
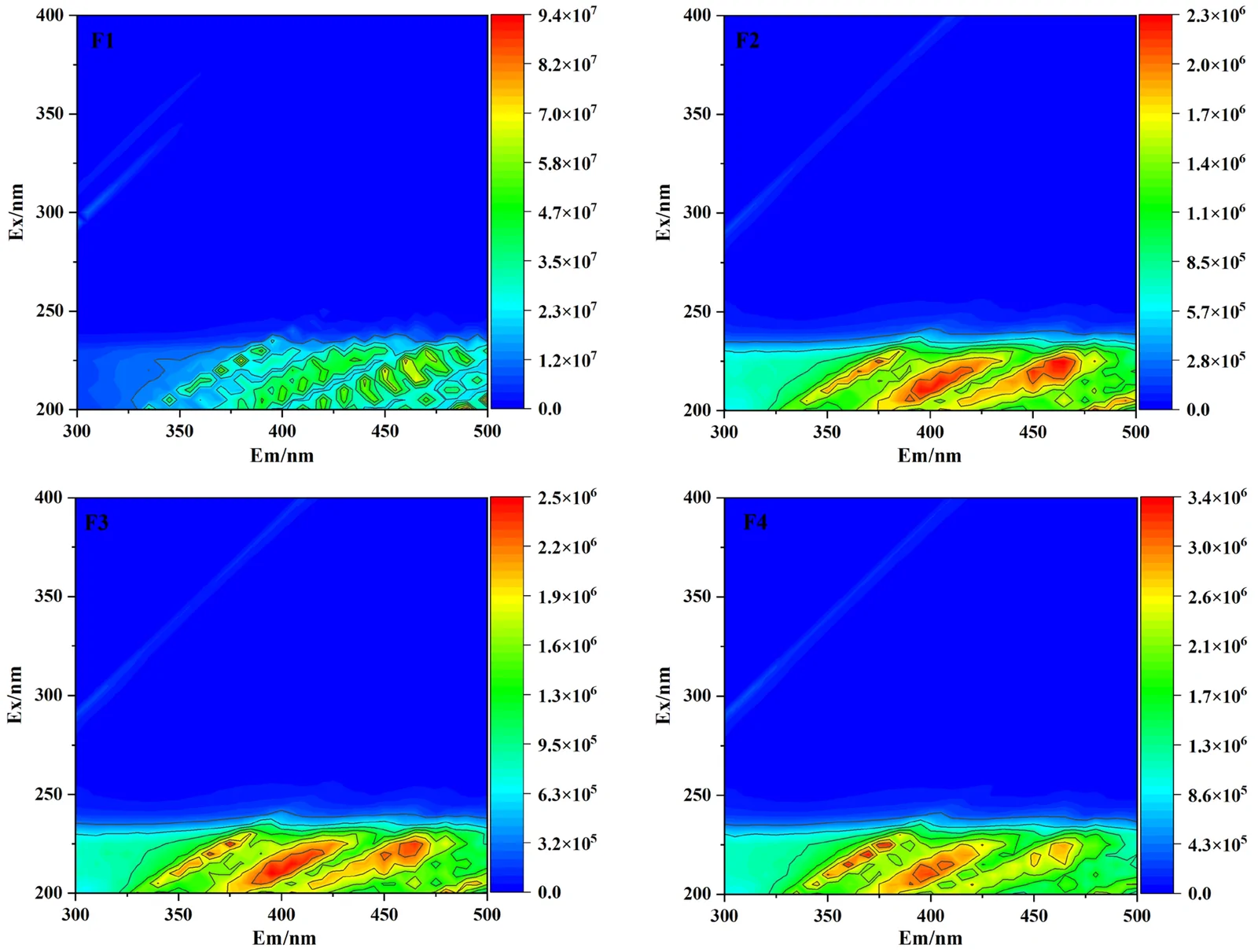
Three-dimensional excitation–emission matrix (EEM) fluorescence spectra of raw forest soil and residues obtained at each stage of sequential extraction. F1, original soil; F2–F4, residues after removal of Ex and HA/FA, HMi, and HMc, respectively. Each panel uses its own intensity scale to highlight peak positions; absolute intensities can be compared through the scale-bar ranges. Those for vegetable and farmland soils are shown in Figures S3 and S4.

EEM spectra (Figure 10) showed that the two fluorescence regions of F1 (Ex/Em ≈ 230–260/360–480 nm and 250–300/420–520 nm) [33] persisted at essentially unchanged positions through F2–F4, whereas the absolute intensity dropped by about two orders of magnitude after the first two extraction steps (from 9.4 × 10^7^ to ~2–3 × 10^6^ a.u.) and then remained at this low residual level. Together with the FTIR and SEM evidence, this indicates that fluorophore-bearing organic matter is released early in the sequence and that the final residues are dominated by the mineral matrix, corroborating the designed stripping order. EEM detects only the water-extractable fluorescent fraction of each residue, so fluorescence intensities were compared after normalization to each sample’s dilution (UV absorbance at 254 nm kept below 0.3). The EEM evidence supports extraction selectivity but does not establish it on its own.

#### 2.1.5. Establishment of Unified Extraction Conditions

Integrating the above single-factor and comprehensive experimental results, the unified extraction condition parameters for each SOM fraction are summarized in Table 4. The extraction completeness of these unified conditions is tested by mass balance in the next section.

### 2.2. Distribution Characteristics of Soil Organic Matter Fractions

Only one composite soil sample, collected from the Datianhe area of Lincang City, Yunnan Province, was used to represent each land-use type. The differences in SOM fraction distribution presented below are therefore observational findings: they verify the applicability of the unified extraction system, but they do not warrant conclusions about how land-use type itself drives these patterns.

The total SOM extraction rates of forest, vegetable and farmland soils were 90.05% ± 1.12%, 91.55% ± 0.97% and 96.97% ± 1.27%, respectively, all above 90%. Forest soil contained high levels of Fe_2_O_3_ (43.1 g kg^−1^) and Al_2_O_3_ (101 g kg^−1^), leading to the lowest recovery. Metal oxides stabilize organic matter via complexation [4]. The large HMr pool (33.65%), together with the 10% unrecovered fraction, points to a substantial share of highly polymerized organic matter in this soil [34]. Vegetable soil possessed the highest organic matter content and CEC, with HA accounting for 48.54% of total SOM; its slightly lower extraction rate may be attributed to the loss of weakly humified organic substances. Farmland soil had the lowest SOM, metal oxide contents, CEC and pH (4.78). Long-term tillage breaks soil aggregates and weakens organo-mineral associations [35], which explains its maximum SOM recovery. Such disparities are consistent with differing SOM stabilization tendencies among the three soils, although the single-composite design does not allow them to be attributed to land use per se.

**Figure 11:**
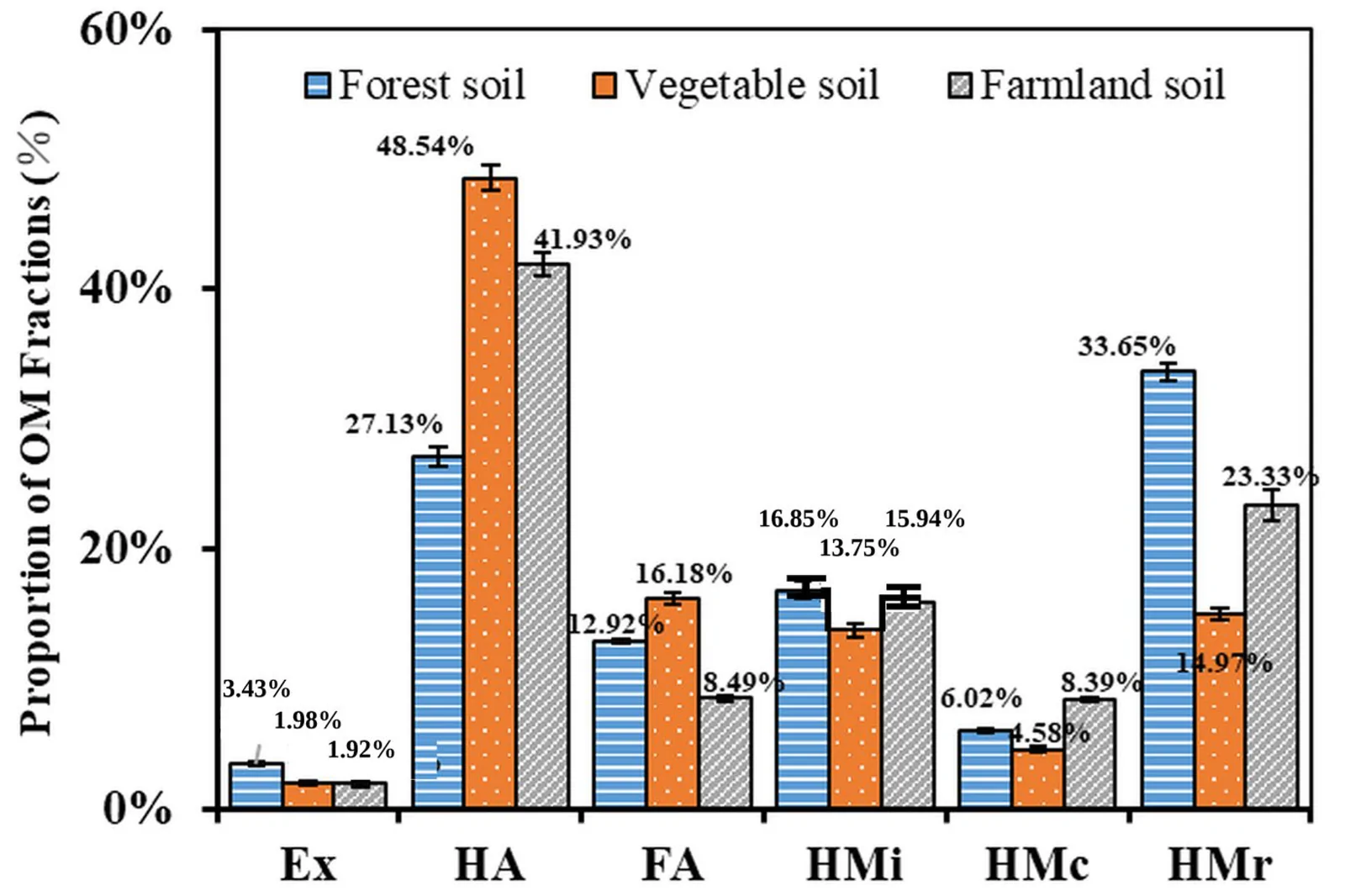
Relative proportions of six soil organic matter fractions under unified extraction conditions. Values are means ± standard deviation (*n* = 3) of the proportion of each fraction to the total extracted organic matter.

Under the unified extraction framework, the three soil types exhibited clearly divergent SOM fraction allocation patterns (Figure 11). Forest soil was dominated by HMr as the primary stable pool (HMr 33.65%; combined HA + FA 40.05%), an allocation compatible with the deep humification and mineral protection expected under long-term, low-disturbance conditions [29,34]. FTIR showed sharply enhanced mineral peaks at the F3–F4 stages. SEM showed densified residue surfaces.

Vegetable soil was dominated by HA as the main active body, with an HA proportion of 48.54%; the combined HMi and HMc accounted for only 18.33%, and HMr accounted for 14.97%. Frequent exogenous inputs and root exudates supply soluble organic components, and competitive adsorption weakens the mineral protection of organic carbon, pointing to a potential “high-input–high-activity–rapid-turnover” state [36,37].

Farmland soil had the lowest total organic matter content (24.5 g kg^−1^), yet the combined HMi and HMc proportion reached 24.33%. Tillage breaks aggregates, exposes organic matter, and allows its secondary adsorption onto Fe and Al oxides and clay minerals, so that a mineral-associated intermediate pool forms between the active and the stable pools [35,38,39].

These results confirm the hypothesis stated in the Introduction. Extraction yields of every fraction entered a plateau once the reagent was strong enough to break the dominant binding bonds, and the plateau regions of the three soils overlapped in each case; the stepwise residues recorded the intended removal order of the fractions; and under the resulting unified conditions the mass balance closed at 90.05–96.97% for all three soils. Fractions obtained under one parameter set therefore carry the same chemical meaning across soils, which is what makes the divergent allocation patterns above comparable at all. How this system relates to existing fractionation schemes, and what it adds to them, is examined in the next section.

### 2.3. Comparison with Existing Fractionation Approaches

The scheme proposed here differs from existing approaches in where the optimization effort is placed. The classical humin fractionation of Pallo [11] and Dou et al. [12] fixed the extraction sequence but left reagent concentrations and reaction times to empirical, soil-specific choice; the IHSS standard procedure [9] standardized the isolation of HA and FA without specifying how parameters should transfer across soil types. Baglieri et al. [28] recovered the residual organic matter after HA/FA removal but did not fractionate humin further, and Weber et al. [21] optimized humin isolation for mineral soils only. None of these schemes reported a mass balance across all fractions, and none tested whether the extraction sequence itself is recorded in the residues. The present framework keeps the classical sequence intact but replaces empirical parameters with conditions drawn from the plateau region shared by three contrasting soils, verifies extraction completeness by mass balance (90.05–96.97%), and documents the sequence step by step in the residues by FTIR, SEM, and EEM.

This design also answers two criticisms raised against operational fractionation. Kleber and Lehmann [19] argued that alkali-extracted humic fractions are invalid proxies for organic matter dynamics because extraction reshapes what is measured; here, residue characterization records which structures each reagent removes, so the operational meaning of every fraction is documented rather than assumed. Olk et al. [17], responding to that criticism, showed that humic fractions accurately represent natural organic matter across diverse settings; the near-complete attenuation of organic markers by the final residue in all three soils supports their conclusion, indicating that cross-contamination under the harmonized conditions is limited. Physical fractionation into particulate and mineral-associated organic matter [20,34] separates pools by size and density and avoids reagent artifacts, but it does not resolve the chemically distinct humin subfractions that control element binding; the two approaches answer complementary questions, and the harmonized chemical scheme provides the fraction-level resolution that physical separation cannot. It should be noted that this comparison rests on published data rather than on a direct side-by-side experiment using the same soils; the claimed advantages of the harmonized conditions therefore concern parameter harmonization and mass-balance documentation, not demonstrated superiority in fraction purity, which awaits head-to-head experimental comparison.

## 3. Materials and Methods

### 3.1. Study Area and Sample Collection

Soil samples were collected from the surface layer (0–20 cm) of different land-use types in the Datianhe area of Lincang City, Yunnan Province, China (23°47′26.00″–23°50′41.48″ N, 99°59′28.84″–100°00′26.00″ E). Representative forest soil, vegetable soil, and farmland soil were selected as the research objects. Samples were collected using a multi-point composite sampling method. After removal of visible impurities, the soils were air-dried at room temperature, ground, passed through a 100-mesh sieve (0.15 mm), thoroughly homogenized, and stored in sealed containers for subsequent analysis. The basic physicochemical properties of the three soils are summarized in Table 5.

Each composite sample was formed by pooling topsoil (0–20 cm, corresponding to the plough/A horizon) collected at three points along an S-shaped transect within one representative plot per land-use type; the subsamples were bulked, homogenized, and reduced by quartering. According to the Chinese Genetic Soil Classification, cross-referenced to the Chinese Soil Taxonomy (CST) and the World Reference Base for Soil Resources (WRB), based on the evidence from the regional soil survey and the acidic, Fe- and Al-oxide-rich, low-CEC character of the samples (Table 5), the forest, vegetable, and farmland soils are classified as red earths (approximately Ferrosols in the CST and Ferralsols in the WRB). The composite design means that each value represents one well-homogenized sample per land-use type, and replication in this study refers to analytical replicates of the composite samples, not to independent field plots.

### 3.2. Experimental Methods

#### 3.2.1. Sequential Extraction Method for Soil Organic Matter Fractions

The full sequence of extraction stages, reagents, and unified conditions is summarized in Table 6. The sequential extraction of soil organic matter was performed following the method of Dou et al. [12]. All extractions were maintained in a thermostatic water-bath shaker at 60 ± 1 °C and a solid-to-liquid ratio of 1:10 (*w*/*v*, 5.0 g soil in 50 mL extractant) unless otherwise stated. After each extraction step, the suspension was centrifuged at 4000 rpm for 15 min. The supernatant was collected for analysis, and 20 mL of deionized water was added to the residue for oscillatory washing for 10 min, followed by centrifugation to discard the wash supernatant. This washing procedure was repeated 2–3 times until the pH of the residue approached neutrality. The 60 °C extraction temperature follows the classical humin fractionation protocol [12] and was adopted to accelerate reagent diffusion and mineral dissolution while remaining below temperatures known to cause appreciable thermal alteration of humic structures. Centrifugation was performed in 100 mL screw-capped centrifuge tubes, which accommodate the full suspension volume (5.0 g soil in 50 mL extractant) and prevent liquid loss when the tubes are opened after centrifugation at 4000 rpm; the hard sediment layer occasionally formed at the top of the tube after centrifugation was loosened with a glass rod, dispersed on a vortex mixer, and inspected visually to confirm that no consolidated sediment remained before the next extractant was introduced. A dedicated water-extraction step for water-soluble organic matter (WSOM) was not included. WSOM typically accounts for a very small share of total SOM and is conventionally characterized as a standalone pool with its own established protocols [18]; the present framework targets the humus fractionation sequence, and adding a water step would introduce an additional operational boundary without changing the humin subfractionation that is the focus of this study. WSOM of the residues was nevertheless monitored by EEM as a diagnostic indicator (Section 3.3).

HA/FA separation: The alkaline extract was centrifuged at 4000 rpm for 15 min to remove mineral particles. The supernatant was acidified with 1 mol L^−1^ HCl to pH 1.0 ± 0.1 and allowed to stand at room temperature for 12 h, then centrifuged at 4000 rpm for 15 min. The precipitate was collected as HA, washed twice with 0.1 mol L^−1^ HCl to remove adsorbed FA, redissolved in 0.1 mol L^−1^ NaOH, and diluted to 50 mL for organic matter content determination. The acid supernatant and washings were combined and filtered through a 0.45 μm cellulose acetate membrane to obtain the FA fraction.

HMi extraction: The residue after HA/FA removal was treated with H_2_SO_4_ at 60 °C for the specified time and concentration (Table 7). After centrifugation at 4000 rpm for 15 min and washing to neutrality, the residue was extracted with NaOH + saturated Na_2_SO_4_ under the same temperature and time conditions. The supernatant was collected after centrifugation and washing. Under the unified conditions, the acid step is 0.5 mol L^−1^ H_2_SO_4_ for 8 h and the alkaline step is 0.5 mol L^−1^ NaOH + saturated Na_2_SO_4_ for 12 h (Table 4).

HMc extraction: The residue after HMi removal was treated with H_2_SO_4_–HF (1:1, *v*/*v*) at 60 °C for the specified time and concentration (Table 7). After centrifugation at 4000 rpm for 15 min and washing to neutrality, the residue was extracted with NaOH + saturated Na_2_SO_4_, followed by centrifugation and washing. Under the unified conditions, the acid step is 1.0 mol L^−1^ H_2_SO_4_ + HF (1:1, *v*/*v*) for 12 h and the alkaline step is 0.5 mol L^−1^ NaOH + saturated Na_2_SO_4_ for 12 h (Table 4).

Following the complete sequential extraction (Ex, HA/FA, HMi, and HMc), the final residues were freeze-dried. Owing to the density difference between organic matter and silicate minerals, the upper dark layer was identified as HMr and the lower gray-white layer as residual mineral (Res). The organic matter contents of the collected solid HMr and Res were determined by the same potassium dichromate–sulfuric acid external heating oxidation–titrimetric method [41], after the separated materials were air-dried, homogenized, and ground to pass a 0.15 mm sieve.

To standardize the separation, scraping was stopped at the visible color boundary between the dark organic layer and the gray-white mineral layer, and the thin interfacial zone was excluded from both fractions to minimize cross-contamination. One trained operator performed all separations, using the visible color boundary as the only endpoint criterion. The separation itself is density-driven stratification formed during freeze-drying, followed by manual scraping; it is not a quantitative density-gradient method. Repeating the full procedure three times on subsamples of the same residue gave an RSD of 2.02–5.06% for HMr mass. This within-operator repeatability does not demonstrate inter-operator reproducibility or fraction purity; operator-independent boundary criteria remain a necessary standardization step.

#### 3.2.2. Optimization of Sequential Extraction Conditions

Based on the parameters listed in Table 7, single-factor experiments and comprehensive experiments (two-factor five-level full factorial design, 5 × 5 = 25 treatment combinations for each soil type) were conducted, with the extraction yield of each SOM fraction (g kg^−1^) as the sole evaluation index. No replicate was set for each treatment combination in the comprehensive experiments to maintain design balance; in the two-factor ANOVA, the interaction term (A × B) was used as the error term, assuming that the interaction between time and concentration was negligible relative to the main effects.

This design follows standard practice for unreplicated two-factor factorials. With 25 combinations per soil, the 24 degrees of freedom split into 4 (time) + 4 (concentration) main-effect terms and 16 interaction terms, and the interaction provides the error estimate. The negligible-interaction assumption is supported by the single-factor experiments, where each factor reached a stable plateau regardless of the other factor’s level, and by the agreement between the ANOVA and range-analysis hierarchies (Section 2.1.2). We therefore use the F-tests only to rank factor importance, not to infer population parameters. In the absence of independent replicates, they cannot provide a definitive statistical justification of the selected parameters; the parameter choice rests primarily on the convergence of the plateau regions across the three soils and on the independent validation recoveries reported below. As an independent check of the selected conditions, validation runs under the unified conditions were performed in triplicate for each soil (the mass-balance experiment of Section 2.2): total SOM recoveries were 90.05% ± 1.12%, 91.55% ± 0.97%, and 96.97% ± 1.27% for the forest, vegetable, and farmland soils, respectively (*n* = 3). The six-fraction distributions under these conditions are those reported in Section 2.2 and Figure 11.

### 3.3. Analytical Methods and Data Processing

The organic matter content in each extract was determined by the potassium dichromate–sulfuric acid external heating oxidation–titrimetric method [41]. Single-factor experiments were performed with three parallel replicates (*n* = 3), and the results are presented as mean values. Matrix-matched blanks containing the corresponding extractants (e.g., NH_4_Ac, NaOH–Na_4_P_2_O_7_, NaOH–Na_2_SO_4_) were analyzed in parallel to correct for salt-induced matrix effects. For HA quantification, the acid-precipitated HA was redissolved in 0.1 mol L^−1^ NaOH and diluted to a fixed volume of 50 mL to ensure consistent volumetric normalization across all samples. The dichromate method measures oxidizable organic carbon, and values are reported as organic matter after applying the Van Bemmelen factor (1.724) and the oxidation-correction coefficient (1.1) specified in NY/T 1121.6-2006 [41]. Each batch was checked with matrix-matched blanks, duplicate analyses (relative percent difference below 5%), and the certified reference soil GBW07403 (GSS-3, yellow-brown earth); measured values, converted to organic carbon (SOM ÷ 1.724), fell within the certified uncertainty interval.

The total SOM extraction rate (*η*, %) was calculated as the ratio of the sum of organic matter contents in all six extracted fractions to the total organic matter content of the original soil determined by the same dichromate method: Because HMr is recovered from the solid residue rather than extracted into solution, η quantifies mass-balance closure of the whole fractionation (extracted plus insoluble organic matter) rather than solution-phase extraction efficiency alone. The solution-phase extraction efficiencies, i.e., ΣCi for the five extracted fractions (Ex, HA, FA, HMi, HMc) relative to C_total_, were 59.75%, 77.84%, and 74.35% for the forest, vegetable, and farmland soils, respectively. Organic matter losses occur at three points: the discarded washing solutions, the interfacial layer excluded during HMr/Res separation, and organic matter retained in the mineral residue (Res organic matter: 3.21, 8.79, 0.34 g kg^−1^); together these account for the unrecovered 3.03–9.95%. The extracted mass of each fraction was calculated from the nominal extractant volume (50 mL) and the measured concentration, so liquid retained in the soil cake after centrifugation is already accounted for in the preceding fraction, and the inter-step washes serve only to remove carry-over of the previous extractant; losses in the discarded washes were not independently quantified, and the uncertainty of η is bounded by the triplicate standard deviations (0.97–1.27%).

(1) $$\begin{array}{c} \eta \left(\%\right)=\frac{\sum_{i=1}^{6}C_{i}}{C_{total}}\times 100\% \end{array}$$

where *C*_i_ represents the organic matter content (g kg^−1^) of each extracted fraction (Ex, HA, FA, HMi, HMc, and HMr), and *C*_total_ represents the total organic matter content (g kg^−1^) of the original soil.

FTIR spectroscopy was performed on a Nicolet iS50 spectrometer (Thermo Fisher Scientific, Waltham, MA, USA) equipped with a DTGS detector using the KBr pellet method (sample:KBr = 1:100, *w*/*w*); scanning range 4000–400 cm^−1^, resolution 4 cm^−1^, 64 scans per sample, with pure KBr background correction [30,31].

SEM was performed on a Quattro S microscope (Thermo Fisher Scientific, Waltham, MA, USA). Freeze-dried residues were Pt-coated (5 nm) and mounted on aluminum stubs (15 kV, 10 mm working distance). Imaging followed the manufacturer’s standard protocol for Pt-coated soil samples.

EEM characterization targeted the water-extractable organic matter of the original soil and residues, serving as an optical indicator of residual fluorescent-active components. For EEM analysis, the original soil (F1) and freeze-dried stepwise extraction residues (F2–F4) were extracted with ultrapure water at a solid-to-liquid ratio of 1:100 (*w*/*v*), shaken at 25 °C for 2 h, centrifuged at 4000 rpm for 15 min, and filtered through a 0.45 μm cellulose acetate membrane. The filtrate was diluted as necessary to maintain UV absorbance at 254 nm below 0.3 to minimize inner-filter effects [18].

EEM was recorded on a Hitachi F-7100 spectrophotometer (Hitachi High-Tech Corporation, Tokyo, Japan) (Ex/Em slit 5 nm; Ex 200–450 nm, step 5 nm; Em 280–550 nm, step 1 nm), with blank subtraction and Rayleigh masking. All analyses were performed in duplicate.

Experimental data were processed and plotted using Microsoft Excel 2019 (Microsoft Corp., Redmond, WA, USA) and Origin 2022 (OriginLab Corp., Northampton, MA, USA). Statistical analyses were performed using SPSS 26.0 (IBM Corp., Armonk, NY, USA).

## 4. Conclusions

We established a harmonized extraction framework for six SOM fractions that applies to three contrasting land-use soils. The six-step sequence runs at 60 °C with a 1:10 solid-to-liquid ratio: a neutral salt displaces Ex, an alkaline–chelate mixture dissolves HA/FA, and two “acid destruction–alkaline extraction” steps release HMi and HMc by selectively disrupting Fe oxides and silicate minerals; the freeze-dried final residue is then split into HMr and the mineral residue by density (Table 4).

FTIR, SEM, and EEM showed that residues evolved from an organic-matter-enriched state to a mineral-skeleton-exposed state as extraction progressed. Organic functional groups weakened stepwise, and the fluorescence intensity fell by about two orders of magnitude to a low residual level, while silicate and Fe–Al mineral signals grew correspondingly. The residue trajectories are therefore consistent with stripping in the intended weak-to-strong order and with limited cross-contamination under the harmonized conditions.

Under the unified extraction conditions, the extraction rates for all three soil types reached or exceeded 90%, indicating adequate mass-balance closure of the unified parameters for the three investigated soils. Under the unified extraction conditions, the three soils showed distinct allocation patterns that can be compared on a common scale: an HMr-dominated pattern in the forest soil, an HA/FA-rich pattern in the vegetable soil, and an HMi/HMc-enriched pattern in the farmland soil. Because all three patterns were obtained under one parameter set, the between-soil differences more likely reflect soil properties than parameter artifacts; whether the corresponding fractions are chemically equivalent across soils awaits molecular-level confirmation.

Several follow-up lines of work emerge. Extending the framework to multiple independent sites across climatic zones will test its robustness against within-site spatial variability and its transferability beyond the study region. Molecular-level techniques such as CP-MAS ^13^C NMR should accompany the present verification, confirming fraction structures independently of extraction temperature and excluding thermal artifacts. The density-based separation of HMr and Res will be standardized with quantitative boundary criteria to make it reproducible across operators and laboratories. And when more than three factors enter the optimization, definitive screening designs or response surface methodologies should keep the search efficient. Should these extensions hold, the framework would approach a general basis for cross-soil SOM fractionation. In addition, applying the harmonized fractions to assess element mobility and retention (e.g., trace metals or germanium) is a promising direction, but such applications require direct element measurements across fractions and are therefore proposed here as future research rather than an outcome of the present study.

## Figures and Tables

**Table 1:** Comprehensive experimental results for Ex extraction in forest soil.

Run	Time (h)	Concentration (mol L^−1^)	OM Content (g kg^−1^)
1	2	0.05	0.0261
2	2	0.1	0.0933
3	2	0.5	0.269
4	2	1.0	0.700
5	2	2.0	0.715
6	5	0.05	0.0369
7	5	0.1	0.105
8	5	0.5	0.597
9	5	1.0	1.29
10	5	2.0	1.35
11	8	0.05	0.0580
12	8	0.1	0.255
13	8	0.5	0.683
14	8	1.0	1.36
15	8	2.0	1.33
16	12	0.05	0.0864
17	12	0.1	0.301
18	12	0.5	0.702
19	12	1.0	1.42
20	12	2.0	1.39
21	24	0.05	0.0928
22	24	0.1	0.305
23	24	0.5	0.696
24	24	1.0	1.40
25	24	2.0	1.38

**Table 2:** Range analysis for Ex extraction in forest soil.

Factor	*k*1	*k*2	*k*3	*k*4	*k*5	*R*
Time (h)	0.361	0.675	0.737	0.779	0.775	0.418
Concentration (mol L^−1^)	0.0600	0.212	0.589	1.23	1.23	1.17

**Table 3:** Analysis of variance (ANOVA) for exchangeable soil organic matter (Ex) extraction in the three soils.

Soil Type	Source	SS	df	MS	*F*	Significance
Forest soil	Time	0.615	4	0.154	9.08	**
	Concentration	6.11	4	1.53	90.3	**
	Error	0.271	16	0.0169		
	Total	7.00	24			
Vegetable soil	Time	2.21	4	0.553	23.1	**
	Concentration	12.2	4	3.05	127	**
	Error	0.384	16	0.0240		
	Total	14.8	24			
Farmland soil	Time	0.284	4	0.0710	26.7	**
	Concentration	0.491	4	0.123	46.2	**
	Error	0.0425	16	0.00266		
	Total	0.818	24			

Note: ** indicates significance at *p* < 0.01 (F-critical (df_1_ = 4, df_2_ = 16, α = 0.01) = 4.77).

**Table 4:** Unified extraction conditions for each SOM fraction.

Step	Fraction	Extractant	Concentration (mol L^−1^)	Extraction Time (h)
1	Ex	NH_4_Ac (pH = 5.0)	1.0	12
2	HA/FA	NaOH + Na_4_P_2_O_7_	1.0 + 0.05	12
3	HMi-1	H_2_SO_4_	0.5	8
4	HMi-2	NaOH + Na_2_SO_4_ (saturated)	0.5 + saturated	12
5	HMc-1	H_2_SO_4_ + HF (1:1, *v*/*v*)	1.0 + 1.0	12
6	HMc-2	NaOH + Na_2_SO_4_ (saturated)	0.5 + saturated	12

Note: HMr and Res are obtained by density separation of the freeze-dried final residue (Section 3.2.1), not by reagent extraction, and are therefore not listed here.

**Table 5:** Physicochemical properties of the soils.

Soil Type	OM (g kg^−1^)	pH	CEC (cmol kg^−1^)	Al_2_O_3_ (g kg^−1^)	Fe_2_O_3_ (g kg^−1^)
Forest soil	44.8	5.02	13.7	101	43.1
Vegetable soil	119	5.72	18.6	85.3	29.0
Farmland soil	24.5	4.78	12.7	35.6	16.3

Note: pH was measured in a 1:2.5 (*w*/*v*) soil–water suspension with a PHS-3E pH meter (INESA Scientific Instrument Co., Ltd., Shanghai, China); CEC denotes the potential CEC determined by exchange with 1 mol L^−1^ ammonium acetate solution at pH 7.0; Al_2_O_3_ and Fe_2_O_3_ are total contents expressed as oxides, determined by inductively coupled plasma optical emission spectrometry after alkali fusion (HJ 974-2018 [40]); organic matter by the potassium dichromate oxidation–titrimetric method [41].

**Table 6:** Overview of the sequential extraction procedure: stages, fractions, reagents, and unified conditions. HMr and Res are separated physically from the final residue, not by reagent extraction.

Stage	Fraction	Reagent	Unified Conditions
1	Ex	NH_4_Ac (pH 5.0)	1.0 mol L^−1^, 12 h
2	HA/FA	NaOH + Na_4_P_2_O_7_	1.0 + 0.05 mol L^−1^, 12 h
3a	HMi (acid step)	H_2_SO_4_	0.5 mol L^−1^, 8 h
3b	HMi (alkaline step)	NaOH + saturated Na_2_SO_4_	0.5 mol L^−1^, 12 h
4a	HMc (acid step)	H_2_SO_4_ + HF (1:1, *v*/*v*)	1.0 mol L^−1^, 12 h
4b	HMc (alkaline step)	NaOH + saturated Na_2_SO_4_	0.5 mol L^−1^, 12 h
5	HMr	Density separation of freeze-dried residue (upper dark layer)	—
6	Res	Density separation of freeze-dried residue (lower gray-white layer)	—

**Table 7:** Factors and levels of the single-factor experiments for extractant concentration and extraction time.

Step	Fraction	Extractant and Concentration Range (mol L^−1^)	Extraction Time Range (h)
1	Ex	0.05, 0.1, 0.5, 1.0, 2.0 mol L^−1^ NH_4_Ac (pH = 5.0)	2, 5, 8, 12, 24
2	HA	0.05, 0.1, 0.5, 1.0, 2.0 mol L^−1^ NaOH + 0.05 mol L^−1^ Na_4_P_2_O_7_	2, 5, 8, 12, 24
3	FA		
4	HMi	0.05, 0.1, 0.5, 1.0, 2.0 mol L^−1^ H_2_SO_4_; 0.05, 0.1, 0.5, 1.0, 2.0 mol L^−1^ NaOH + saturated Na_2_SO_4_	2, 5, 8, 12, 24
5	HMc	0.05, 0.1, 0.5, 1.0, 2.0 mol L^−1^ H_2_SO_4_ + HF (1:1, *v*/*v*); 0.05, 0.1, 0.5, 1.0, 2.0 mol L^−1^ NaOH + saturated Na_2_SO_4_	2, 5, 8, 12, 24
6	HMr	Density separation of the freeze-dried final residue (upper layer = HMr, lower layer = Res)	—
7	Res		

Note: HF concentration was set equal to H_2_SO_4_ concentration in each treatment. HA and FA are co-extracted in a single alkaline step and separated afterward; they are listed as separate rows because their yields were optimized individually. HMr and Res are obtained by density separation and were not included in the single-factor optimization.

## Data Availability

The data presented in this study are available on request from the corresponding author. The raw data supporting the comprehensive experimental results are provided in Supplementary Materials (Tables S1–S58 and Figures S1–S4).

## Supplementary Materials

The following supporting information can be downloaded at: https://www.mdpi.com/article/10.3390/molecules31213880/s1, Tables S1–S58: Comprehensive experimental, range analysis, and ANOVA results for all fraction extractions; Figure S1: FTIR spectra of the farmland soil after sequential extraction; Figure S2: SEM micrographs of the farmland soil after sequential extraction; Figures S3 and S4: EEM fluorescence spectra of the vegetable and farmland soils.

## Abbreviations

The following abbreviations are used in this manuscript: SOM, soil organic matter; Ex, exchangeable soil organic matter; HA, humic acid; FA, fulvic acid; HM, humin; HMi, iron-bound humin; HMc, clay-bound humin; HMr, insoluble humin; Res, residual mineral; CEC, cation exchange capacity; FTIR, Fourier-transform infrared spectroscopy; SEM, scanning electron microscopy; EEM, three-dimensional excitation–emission matrix; ANOVA, analysis of variance; IHSS, International Humic Substances Society.

## References

[1] Schmidt M.W.I., Torn M.S., Abiven S., Dittmar T., Guggenberger G., Janssens I.A., Kleber M., Kögel-Knabner I., Lehmann J., Manning D.A.C. (2011). Persistence of Soil Organic Matter as an Ecosystem Property. Nature.

[2] Lal R. (2004). Soil Carbon Sequestration Impacts on Global Climate Change and Food Security. Science.

[3] Tipping E., Somerville C.J., Luster J. (2016). The C:N:P:S Stoichiometry of Soil Organic Matter. Biogeochemistry.

[4] Kaiser K., Guggenberger G. (2003). Mineral Surfaces and Soil Organic Matter. Eur. J. Soil Sci..

[5] Kögel-Knabner I., Guggenberger G., Kleber M., Kandeler E., Kalbitz K., Scheu S., Eusterhues K., Leinweber P. (2008). Organo-Mineral Associations in Temperate Soils: Integrating Biology, Mineralogy and Organic Matter Chemistry. J. Plant Nutr. Soil Sci..

[6] Lehmann J., Kleber M. (2015). The Contentious Nature of Soil Organic Matter. Nature.

[7] Milne C.J., Kinniburgh D.G., van Riemsdijk W.H., Tipping E. (2003). Generic NICA-Donnan Model Parameters for Metal-Ion Binding by Humic Substances. Environ. Sci. Technol..

[8] Senesi N., Loffredo E., Sparks D.L. (1999). The Chemistry of Soil Organic Matter. Soil Physical Chemistry.

[9] Swift R.S., Sparks D.L. (1996). Organic Matter Characterization. Methods of Soil Analysis: Part 3 Chemical Methods.

[10] Hayes M.H.B., Roulia M. (2025). Relevance of Organic Matter Compositions, Structures and Associations to Soil Aggregates and to Sustainable Productivity. Agronomy.

[11] Pallo F.J.P., Mulongoy K., Merckx R. (1993). Evolution of Organic Matter in Some Soils under Shifting Cultivation Practices in Burkina Faso. Soil Organic Matter Dynamics and Sustainability of Tropical Agriculture.

[12] Dou S., Xiao Y.C., Zhang J.J. (2006). Preliminary Study on the Quantity and Structural Characteristics of Humin Fractions in Soil. Acta Pedol. Sin..

[13] Fan C.Y., Xie X.H., Yan A.C., Zhang J.J. (2018). Structure and Cu(II) Adsorption of Soil Humin. Acta Pedol. Sin..

[14] Fan B., Tang M., Yao L., Zhang A., Yin H., Yang W., Ma Z., Xiang W., Bao Z. (2021). Germanium Fractions in Typical Paddy Soil and Its Interaction With Humic Substances. Environ. Sci. Pollut. Res..

[15] Zhang J., Yin H.L., Wang H., Xu L., Samuel B., Chang J.J., Liu F., Chen H.H. (2019). Molecular Structure-Reactivity Correlations of Humic Acid and Humin Fractions From a Typical Black Soil for Hexavalent Chromium Reduction. Sci. Total Environ..

[16] Nebbioso A., Piccolo A. (2011). Basis of a Humeomics Science: Chemical Fractionation and Molecular Characterization of Humic Biosuprastructures. Biomacromolecules.

[17] Olk D.C., Bloom P.R., De Nobili M., Chen Y., McKnight D.M., Wells M.J.M., Weber J. (2019). Using Humic Fractions to Understand Natural Organic Matter Processes in Soil and Water: Selected Studies and Applications. J. Environ. Qual..

[18] Fasching C., Boodoo K.S., Feld-Golinski A., Foroushani M.A., Chifflard P. (2026). Extraction Method Shapes Soil Water-Soluble Organic Matter Composition as Revealed by Absorbance, Fluorescence, and Parallel Factor Analysis (PARAFAC). Sci. Rep..

[19] Kleber M., Lehmann J. (2019). Humic Substances Extracted by Alkali Are Invalid Proxies for the Dynamics and Functions of Organic Matter in Terrestrial and Aquatic Ecosystems. J. Environ. Qual..

[20] von Lützow M., Kögel-Knabner I., Ekschmitt K., Flessa H., Guggenberger G., Matzner E., Marschner B. (2007). SOM Fractionation Methods: Relevance to Functional Pools and to Stabilization Mechanisms. Soil Biol. Biochem..

[21] Weber J., Jamroz E., Kocowicz A., Debicka M., Bekier J., Cwielag-Piasecka I., Ukalska-Jaruga A., Mielnik L., Bejger R., Jerzykiewicz M. (2022). Optimized Isolation Method of Humin Fraction From Mineral Soil Material. Environ. Geochem. Health.

[22] Ponge J.F. (2003). Humus Forms in Terrestrial Ecosystems: A Framework to Biodiversity. Soil Biol. Biochem..

[23] Guimarães D.V., Gonzaga M.I.S., da Silva T.O., da Silva T.L., Dias N.D., Matias M.I.S. (2013). Soil Organic Matter Pools and Carbon Fractions in Soil Under Different Land Uses. Soil Tillage Res..

[24] Benbi D.K., Brar K., Toor A.S., Sharma S. (2015). Sensitivity of Labile Soil Organic Carbon Pools to Long-Term Fertilizer, Straw and Manure Management in a rice–wheat System. Pedosphere.

[25] Zhu X., Shen Y.X., Yuan X., Singh A.K., Jin L.Y., Yang B., Yuan C., Jiang X.J., Liu W.J. (2025). Divergent Responses of Soil Aggregate-Associated Organic Carbon Fractions and Carbon Flow Pathways to Land-Use Changes in Karst Ecosystems: Insights From δ13C Signature. Catena.

[26] Emde D., Sakhaee A., Poeplau C., Don A., Scherstjanoi M., Wellbrock N., Schneider F. (2025). Site-Specific Drivers of Land-Use Change Effects on Organic Carbon in German Agriculture and Forest Soils. Glob. Change Biol..

[27] Yang X.M., Zhang J., Mostofa K.M.G., Mohinuzzaman M., Teng H.H., Senesi N., Senesi G.S., Yuan J., Liu Y., Li S.L. (2025). Solubility Characteristics of Soil Humic Substances as a Function of pH: Mechanisms and Biogeochemical Perspectives. Biogeosciences.

[28] Baglieri A., Ioppolo A., Negre M., Gennari M. (2007). A Method for Isolating Soil Organic Matter After the Extraction of Humic and Fulvic Acids. Org. Geochem..

[29] von Lützow M., Kögel-Knabner I., Ekschmitt K., Matzner E., Guggenberger G., Marschner B., Flessa H. (2006). Stabilization of Organic Matter in Temperate Soils: Mechanisms and Their Relevance Under Different Soil Conditions—A Review. Eur. J. Soil Sci..

[30] Wu Y., Geng J., Fang H.J., Cheng S.L. (2025). Research Progress on Fourier Transform Infrared Spectroscopy in Chemical Composition Analysis of Soil Organic Matter. Spectrosc. Spectr. Anal..

[31] Song D.S., Sheng H., Zhou Q., Zhang Y.Z., Wang C.H., Zhou P. (2016). Mid-Infrared Absorption Spectral Characteristics of Soils Developed From Different Parent Materials. Chin. J. Soil Sci..

[32] Ukalska-Jaruga A., Bejger R., Debaene G., Smreczak B. (2021). Characterization of Soil Organic Matter Individual Fractions (Fulvic Acids, Humic Acids, and Humins) by Spectroscopic and Electrochemical Techniques in Agricultural Soils. Agronomy.

[33] Tang C.R., Hou J.W., Li J., Yu H.B., Nie L. (2025). Analysis of Humic Acid Components and Source Characteristics in Riparian Soil Based on Three-Dimensional Fluorescence and Absolute Principal Component Score. J. Environ. Eng. Technol..

[34] Six J., Conant R.T., Paul E.A., Paustian K. (2002). Stabilization Mechanisms of Soil Organic Matter: Implications for C-Saturation of Soils. Plant Soil.

[35] Pan B., Tao S., Dawson R.W., Xing B.S. (2010). Formation of Organo-Mineral Complexes as Affected by Particle Size, pH, and Dry-Wet Cycles. Aust. J. Soil Res..

[36] Keiluweit M., Bougoure J.J., Nico P.S., Pett-Ridge J., Weber P.K., Kleber M. (2015). Mineral Protection of Soil Carbon Counteracted by Root Exudates. Nat. Clim. Chang..

[37] Li H., Bölscher T., Winnick M., Tfaily M.M., Cardon Z.G., Kästner M. (2021). Simple Plant and Microbial Exudates Destabilize Mineral-Associated Organic Matter via Multiple Pathways. Environ. Sci. Technol..

[38] Sokol N.W., Whalen E.D., Jilling A., Kallenbach C., Pett-Ridge J., Georgiou K. (2022). Global Distribution, Formation and Fate of Mineral-Associated Soil Organic Matter Under a Changing Climate: A Trait-Based Perspective. Funct. Ecol..

[39] Weber J., Jamroz E., Mielnik L., Spaccini R., Kocowicz A., Cwielag-Piasecka I., Jerzykiewicz M., Parylak D., Debicka M. (2024). Changes in Soil Humin Macromolecular Structure Resulting From Long-Term Catch Cropping. Molecules.

[40] (2018). Soil and Sediment—Determination of 11 Elements—Alkaline Fusion and Inductively Coupled Plasma Optical Emission Spectrometry.

[41] (2006). Soil Testing—Part 6: Method for Determination of Soil Organic Matter.

