# Supplementary material for: Harmonized Sequential Extraction Parameters for Soil Organic Matter Fractions Across Contrasting Land-Use Soils: Optimization and Mass-Balance Assessment
Source: Molecules. 2026 Oct 27;31(21):3880. doi: 10.3390/molecules31213880 (PMC13765338; doi:10.3390/molecules31213880)
Supplement: Supplementary file 1 [file molecules-31-03880-s001.zip › molecules-4632003-supplementary.pdf]

Harmonized Sequential Extraction Parameters for Soil Organic Matter Fractions across Contrasting Land-Use Soils: Optimization and Mass-Balance Assessment.

Supplementary Materials

Table S1. Comprehensive experimental results for exchangeable (Ex) extraction in vegetable soil.

Run	Time (h)	Concentration (mol L^{-1})	OM content (g kg^{-1})
1	2	0.05	0.872
2	2	0.1	0.970
3	2	0.5	1.58
4	2	1.0	1.98
5	2	2.0	2.01
6	5	0.05	0.955
7	5	0.1	1.03
8	5	0.5	1.91
9	5	1.0	2.51
10	5	2.0	2.53
11	8	0.05	1.11
12	8	0.1	1.26
13	8	0.5	2.03
14	8	1.0	2.77
15	8	2.0	2.74
16	12	0.05	1.34
17	12	0.1	1.34
18	12	0.5	2.41
19	12	1.0	3.26
20	12	2.0	3.10
21	24	0.05	1.30
22	24	0.1	1.33
23	24	0.5	2.37
24	24	1.0	3.11
25	24	2.0	3.05

Table S2. Range analysis for exchangeable (Ex) extraction in vegetable soil.

Factor	k1	k2	k3	k4	k5	R
Time (h)	1.48	1.79	1.98	2.29	2.23	0.810
Concentration (mol L^{-1})	1.11	1.19	2.06	2.73	2.69	1.61

Table S3. Comprehensive experimental results for exchangeable (Ex) extraction in farmland soil.

Run	Time (h)	Concentration (mol L^{-1})	OM content (g kg^{-1})
1	2	0.05	0.0520
2	2	0.1	0.186
3	2	0.5	0.237
4	2	1.0	0.321
5	2	2.0	0.316
6	5	0.05	0.0630
7	5	0.1	0.251
8	5	0.5	0.336
9	5	1.0	0.362
10	5	2.0	0.356
11	8	0.05	0.0670
12	8	0.1	0.294
13	8	0.5	0.395
14	8	1.0	0.458
15	8	2.0	0.454
16	12	0.05	0.177
17	12	0.1	0.391
18	12	0.5	0.456
19	12	1.0	0.697
20	12	2.0	0.676
21	24	0.05	0.234
22	24	0.1	0.408
23	24	0.5	0.466
24	24	1.0	0.679
25	24	2.0	0.634

Table S4. Range analysis for exchangeable (Ex) extraction in farmland soil.

Factor	k1	k2	k3	k4	k5	R
Time (h)	0.222	0.274	0.334	0.479	0.484	0.262
Concentration (mol L^{-1})	0.119	0.306	0.378	0.503	0.487	0.385

Table S5. Comprehensive experimental results for humic acid (HA) extraction in forest soil.

Run	Time (h)	Concentration (mol L^{-1})	OM content (g kg^{-1})
1	2	0.05	5.45
2	2	0.1	6.62
3	2	0.5	7.24
4	2	1.0	7.99
5	2	2.0	8.00
6	5	0.05	6.94
7	5	0.1	8.43
8	5	0.5	9.22
9	5	1.0	9.91
10	5	2.0	9.19
11	8	0.05	7.58
12	8	0.1	9.21
13	8	0.5	10.1
14	8	1.0	11.4
15	8	2.0	11.1
16	12	0.05	8.16
17	12	0.1	9.91
18	12	0.5	10.8
19	12	1.0	11.7
20	12	2.0	11.5
21	24	0.05	8.13
22	24	0.1	10.1
23	24	0.5	10.7
24	24	1.0	11.5
25	24	2.0	11.4

Table S6. Range analysis for humic acid (HA) extraction in forest soil.

Factor	k1	k2	k3	k4	k5	R
Time (h)	7.06	8.74	9.87	10.4	10.4	3.34
Concentration (mol L^{-1})	7.25	8.85	9.62	10.5	10.2	3.23

Table S7. Analysis of variance (ANOVA) for humic acid (HA) extraction in forest soil.

Source	SS	df	MS	F	Significance
Time	40.0	4	10.0	150	**
Concentration	33.9	4	8.47	127	**
Error	1.07	16	0.0667		
Total	75.0	24			

Note: ** indicates significance at $p < 0.01$ (F-critical ($df_1 = 4$, $df_2 = 16$, $\alpha = 0.01$) = 4.77).

Table S8. Comprehensive experimental results for humic acid (HA) extraction in vegetable soil.

Run	Time (h)	Concentration (mol L^{-1})	OM content (g kg^{-1})
1	2	0.05	17.3
2	2	0.1	24.8
3	2	0.5	32.8
4	2	1.0	32.9
5	2	2.0	33.1
6	5	0.05	21.8
7	5	0.1	31.2
8	5	0.5	41.4
9	5	1.0	41.4
10	5	2.0	40.7
11	8	0.05	24.5
12	8	0.1	35.1
13	8	0.5	45.3
14	8	1.0	46.5
15	8	2.0	46.2
16	12	0.05	31.5
17	12	0.1	42.6
18	12	0.5	56.8
19	12	1.0	57.2
20	12	2.0	57.0
21	24	0.05	31.0
22	24	0.1	42.0
23	24	0.5	56.2
24	24	1.0	57.1
25	24	2.0	56.9

Table S9. Range analysis for humic acid (HA) extraction in vegetable soil.

Factor	k1	k2	k3	k4	k5	R
Time (h)	28.2	35.3	39.5	49.0	48.6	20.8
Concentration (mol L^{-1})	25.2	35.1	46.5	47.0	46.8	21.8

Table S10. Analysis of variance (ANOVA) for humic acid (HA) extraction in vegetable soil.

Source	SS	df	MS	F	Significance
Time	1590	4	397	95.7	**
Concentration	1900	4	475	114	**
Error	66.4	16	4.15		
Total	3550	24			

Note: ** indicates significance at $p < 0.01$ (F-critical ($df_1 = 4$, $df_2 = 16$, $\alpha = 0.01$) = 4.77).

Table S11. Comprehensive experimental results for humic acid (HA) extraction in farmland soil.

Run	Time (h)	Concentration (mol L^{-1})	OM content (g kg^{-1})
1	2	0.05	4.20
2	2	0.1	4.76
3	2	0.5	6.71
4	2	1.0	7.41
5	2	2.0	7.41
6	5	0.05	6.32
7	5	0.1	7.00
8	5	0.5	8.15
9	5	1.0	8.82
10	5	2.0	8.84
11	8	0.05	7.06
12	8	0.1	7.82
13	8	0.5	9.11
14	8	1.0	10.5
15	8	2.0	10.2
16	12	0.05	7.43
17	12	0.1	8.23
18	12	0.5	9.59
19	12	1.0	10.6
20	12	2.0	10.4
21	24	0.05	7.38
22	24	0.1	8.19
23	24	0.5	9.54
24	24	1.0	10.4
25	24	2.0	10.4

Table S12. Range analysis for humic acid (HA) extraction in farmland soil.

Factor	k1	k2	k3	k4	k5	R
Time (h)	6.10	7.82	8.95	9.25	9.19	3.15
Concentration (mol L^{-1})	6.48	7.20	8.62	9.56	9.45	3.08

Table S13. Analysis of variance (ANOVA) for humic acid (HA) extraction in farmland soil.

Source	SS	df	MS	F	Significance
Time	35.9	4	8.97	245	**
Concentration	37.7	4	9.42	257	**
Error	0.587	16	0.0367		
Total	74.2	24			

Note: ** indicates significance at $p < 0.01$ (F-critical ($df_1 = 4$, $df_2 = 16$, $\alpha = 0.01$) = 4.77).

Table S14. Comprehensive experimental results for fulvic acid (FA) extraction in forest soil.

Run	Time (h)	Concentration (mol L^{-1})	OM content (g kg^{-1})
1	2	0.05	2.69
2	2	0.1	3.26
3	2	0.5	3.57
4	2	1.0	3.84
5	2	2.0	3.94
6	5	0.05	3.42
7	5	0.1	4.15
8	5	0.5	4.54
9	5	1.0	4.88
10	5	2.0	4.56
11	8	0.05	3.74
12	8	0.1	4.54
13	8	0.5	4.96
14	8	1.0	5.47
15	8	2.0	5.45
16	12	0.05	4.02
17	12	0.1	4.88
18	12	0.5	5.34
19	12	1.0	6.12
20	12	2.0	6.02
21	24	0.05	4.02
22	24	0.1	4.91
23	24	0.5	5.33
24	24	1.0	5.93
25	24	2.0	5.94

Table S15. Range analysis for fulvic acid (FA) extraction in forest soil.

Factor	k1	k2	k3	k4	k5	R
Time (h)	3.46	4.31	4.83	5.27	5.23	1.82
Concentration (mol L^{-1})	3.58	4.35	4.75	5.25	5.18	1.67

Table S16. Analysis of variance (ANOVA) for fulvic acid (FA) extraction in forest soil.

Source	SS	df	MS	F	Significance
Time	11.4	4	2.86	91.0	**
Concentration	9.47	4	2.37	75.3	**
Error	0.503	16	0.0314		
Total	21.4	24			

Note: ** indicates significance at $p < 0.01$ (F-critical ($df_1 = 4$, $df_2 = 16$, $\alpha = 0.01$) = 4.77).

Table S17. Comprehensive experimental results for fulvic acid (FA) extraction in vegetable soil.

Run	Time (h)	Concentration (mol L^{-1})	OM content (g kg^{-1})
1	2	0.05	5.77
2	2	0.1	8.26
3	2	0.5	11.0
4	2	1.0	10.7
5	2	2.0	10.0
6	5	0.05	7.28
7	5	0.1	10.4
8	5	0.5	13.5
9	5	1.0	12.8
10	5	2.0	12.9
11	8	0.05	8.17
12	8	0.1	11.7
13	8	0.5	15.1
14	8	1.0	16.5
15	8	2.0	16.4
16	12	0.05	10.5
17	12	0.1	14.2
18	12	0.5	16.6
19	12	1.0	17.5
20	12	2.0	17.2
21	24	0.05	10.2
22	24	0.1	14.0
23	24	0.5	16.5
24	24	1.0	17.5
25	24	2.0	17.1

Table S18. Range analysis for fulvic acid (FA) extraction in vegetable soil.

Factor	k1	k2	k3	k4	k5	R
Time (h)	9.14	11.4	13.6	15.2	15.1	6.07
Concentration (mol L^{-1})	8.39	11.7	14.5	15.0	14.7	6.61

Table S19. Analysis of variance (ANOVA) for fulvic acid (FA) extraction in vegetable soil.

Source	SS	df	MS	F	Significance
Time	135	4	33.7	75.3	**
Concentration	161	4	40.2	89.8	**
Error	7.16	16	0.448		
Total	303	24			

Note: ** indicates significance at $p < 0.01$ (F-critical ($df_1 = 4$, $df_2 = 16$, $\alpha = 0.01$) = 4.77).

Table S20. Comprehensive experimental results for fulvic acid (FA) extraction in farmland soil.

Run	Time (h)	Concentration (mol L^{-1})	OM content (g kg^{-1})
1	2	0.05	1.12
2	2	0.1	1.34
3	2	0.5	1.68
4	2	1.0	1.82
5	2	2.0	1.87
6	5	0.05	1.58
7	5	0.1	1.75
8	5	0.5	2.04
9	5	1.0	2.27
10	5	2.0	2.26
11	8	0.05	1.77
12	8	0.1	1.96
13	8	0.5	2.28
14	8	1.0	2.55
15	8	2.0	2.58
16	12	0.05	1.86
17	12	0.1	2.16
18	12	0.5	2.40
19	12	1.0	2.75
20	12	2.0	2.67
21	24	0.05	1.82
22	24	0.1	2.11
23	24	0.5	2.36
24	24	1.0	2.66
25	24	2.0	2.67

Table S21. Range analysis for fulvic acid (FA) extraction in farmland soil.

Factor	k1	k2	k3	k4	k5	R
Time (h)	1.57	1.98	2.23	2.37	2.32	0.801
Concentration (mol L^{-1})	1.63	1.86	2.15	2.41	2.41	0.785

Table S22. Analysis of variance (ANOVA) for fulvic acid (FA) extraction in farmland soil.

Source	SS	df	MS	F	Significance
Time	2.18	4	0.545	308	**
Concentration	2.38	4	0.596	337	**
Error	0.0283	16	0.00177		
Total	4.59	24			

Note: ** indicates significance at $p < 0.01$ (F-critical ($df_1 = 4$, $df_2 = 16$, $\alpha = 0.01$) = 4.77).

Table S23. Comprehensive experimental results for iron-bound humin (HMi-1) acid extraction in forest soil.

Run	Time (h)	Concentration (mol L^{-1})	OM content (g kg^{-1})
1	2	0.05	1.57
2	2	0.1	2.87
3	2	0.5	3.23
4	2	1.0	3.25
5	2	2.0	3.26
6	5	0.05	2.05
7	5	0.1	3.75
8	5	0.5	5.08
9	5	1.0	4.95
10	5	2.0	4.95
11	8	0.05	2.42
12	8	0.1	4.42
13	8	0.5	5.83
14	8	1.0	5.77
15	8	2.0	5.83
16	12	0.05	2.67
17	12	0.1	4.60
18	12	0.5	5.68
19	12	1.0	5.60
20	12	2.0	5.52
21	24	0.05	2.83
22	24	0.1	4.62
23	24	0.5	5.62
24	24	1.0	5.52
25	24	2.0	5.51

Table S24. Range analysis for iron-bound humin (HMi-1) acid extraction in forest soil.

Factor	k1	k2	k3	k4	k5	R
Time (h)	2.84	4.16	4.85	4.82	4.82	2.02
Concentration (mol L^{-1})	2.31	4.05	5.09	5.02	5.01	2.78

Table S25. Analysis of variance (ANOVA) for iron-bound humin (HMi-1) acid extraction in forest soil.

Source	SS	df	MS	F	Significance
Time	15.0	4	3.75	42.3	**
Concentration	28.4	4	7.10	80.2	**
Error	1.42	16	0.0885		
Total	44.8	24			

Note: ** indicates significance at $p < 0.01$ (F-critical ($df_1 = 4$, $df_2 = 16$, $\alpha = 0.01$) = 4.77).

Table S26. Comprehensive experimental results for iron-bound humin (HMi-1) acid extraction in vegetable soil.

Run	Time (h)	Concentration (mol L^{-1})	OM content (g kg^{-1})
1	2	0.05	3.39
2	2	0.1	4.00
3	2	0.5	4.54
4	2	1.0	4.61
5	2	2.0	4.69
6	5	0.05	4.11
7	5	0.1	4.85
8	5	0.5	6.53
9	5	1.0	6.47
10	5	2.0	6.48
11	8	0.05	4.52
12	8	0.1	5.60
13	8	0.5	7.55
14	8	1.0	7.33
15	8	2.0	7.33
16	12	0.05	4.54
17	12	0.1	5.71
18	12	0.5	7.43
19	12	1.0	7.23
20	12	2.0	7.24
21	24	0.05	4.73
22	24	0.1	5.77
23	24	0.5	7.39
24	24	1.0	7.21
25	24	2.0	7.21

Table S27. Range analysis for iron-bound humin (HMi-1) acid extraction in vegetable soil.

Factor	k1	k2	k3	k4	k5	R
Time (h)	4.25	5.69	6.46	6.43	6.46	2.22
Concentration (mol L^{-1})	4.26	5.18	6.69	6.57	6.59	2.43

Table S28. Analysis of variance (ANOVA) for iron-bound humin (HMi-1) acid extraction in vegetable soil.

Source	SS	df	MS	F	Significance
Time	18.4	4	4.61	44.0	**
Concentration	23.8	4	5.94	56.7	**
Error	1.68	16	0.105		
Total	43.9	24			

Note: ** indicates significance at $p < 0.01$ (F-critical ($df_1 = 4$, $df_2 = 16$, $\alpha = 0.01$) = 4.77).

Table S29. Comprehensive experimental results for iron-bound humin (HMi-1) acid extraction in farmland soil.

Run	Time (h)	Concentration (mol L^{-1})	OM content (g kg^{-1})
1	2	0.05	1.17
2	2	0.1	1.20
3	2	0.5	2.22
4	2	1.0	2.14
5	2	2.0	2.21
6	5	0.05	1.42
7	5	0.1	2.06
8	5	0.5	2.86
9	5	1.0	2.75
10	5	2.0	2.83
11	8	0.05	1.87
12	8	0.1	2.31
13	8	0.5	3.37
14	8	1.0	3.11
15	8	2.0	3.08
16	12	0.05	1.76
17	12	0.1	2.23
18	12	0.5	3.36
19	12	1.0	3.13
20	12	2.0	3.09
21	24	0.05	1.76
22	24	0.1	2.21
23	24	0.5	3.32
24	24	1.0	3.15
25	24	2.0	3.08

Table S30. Range analysis for iron-bound humin (HMi-1) acid extraction in farmland soil.

Factor	k1	k2	k3	k4	k5	R
Time (h)	1.79	2.38	2.75	2.72	2.70	0.963
Concentration (mol L^{-1})	1.59	2.00	3.02	2.86	2.86	1.43

Table S31. Analysis of variance (ANOVA) for iron-bound humin (HMi-1) acid extraction in farmland soil.

Source	SS	df	MS	F	Significance
Time	3.34	4	0.834	77.0	**
Concentration	7.98	4	1.99	184	**
Error	0.173	16	0.0108		
Total	11.5	24			

Note: ** indicates significance at $p < 0.01$ (F-critical ($df_1 = 4$, $df_2 = 16$, $\alpha = 0.01$) = 4.77).

Table S32. Comprehensive experimental results for iron-bound humin (HMi-2) alkaline extraction in forest soil.

Run	Time (h)	Concentration (mol L^{-1})	OM content (g kg^{-1})
1	2	0.05	0.0497
2	2	0.1	0.349
3	2	0.5	0.836
4	2	1.0	0.841
5	2	2.0	0.943
6	5	0.05	0.0603
7	5	0.1	0.424
8	5	0.5	1.19
9	5	1.0	1.17
10	5	2.0	1.12
11	8	0.05	0.0674
12	8	0.1	0.474
13	8	0.5	1.32
14	8	1.0	1.22
15	8	2.0	1.21
16	12	0.05	0.0810
17	12	0.1	0.499
18	12	0.5	1.36
19	12	1.0	1.26
20	12	2.0	1.26
21	24	0.05	0.0825
22	24	0.1	0.508
23	24	0.5	1.34
24	24	1.0	1.20
25	24	2.0	1.22

Table S33. Range analysis for iron-bound humin (HMi-2) alkaline extraction in forest soil.

Factor	k1	k2	k3	k4	k5	R
Time (h)	0.604	0.792	0.857	0.892	0.869	0.288
Concentration (mol L^{-1})	0.0682	0.451	1.21	1.14	1.15	1.14

Table S34. Analysis of variance (ANOVA) for iron-bound humin (HMi-2) alkaline extraction in forest soil.

Source	SS	df	MS	F	Significance
Time	0.275	4	0.0688	9.83	**
Concentration	5.30	4	1.33	189	**
Error	0.112	16	0.00700		
Total	5.69	24			

Note: ** indicates significance at $p < 0.01$ (F-critical ($df_1 = 4$, $df_2 = 16$, $\alpha = 0.01$) = 4.77).

Table S35. Comprehensive experimental results for iron-bound humin (HMi-2) alkaline extraction in vegetable soil.

Run	Time (h)	Concentration (mol L^{-1})	OM content (g kg^{-1})
1	2	0.05	0.445
2	2	0.1	0.891
3	2	0.5	2.17
4	2	1.0	2.17
5	2	2.0	2.20
6	5	0.05	1.54
7	5	0.1	1.70
8	5	0.5	5.15
9	5	1.0	5.18
10	5	2.0	5.12
11	8	0.05	1.96
12	8	0.1	2.57
13	8	0.5	7.28
14	8	1.0	7.02
15	8	2.0	7.05
16	12	0.05	2.06
17	12	0.1	2.71
18	12	0.5	7.64
19	12	1.0	7.59
20	12	2.0	7.44
21	24	0.05	2.13
22	24	0.1	2.78
23	24	0.5	7.43
24	24	1.0	7.31
25	24	2.0	7.32

Table S36. Range analysis for iron-bound humin (HMi-2) alkaline extraction in vegetable soil.

Factor	k1	k2	k3	k4	k5	R
Time (h)	1.58	3.74	5.17	5.49	5.40	3.91
Concentration (mol L^{-1})	1.63	2.13	5.93	5.85	5.83	4.31

Table S37. Analysis of variance (ANOVA) for iron-bound humin (HMi-2) alkaline extraction in vegetable soil.

Source	SS	df	MS	F	Significance
Time	55.5	4	13.9	19.7	**
Concentration	96.3	4	24.1	34.1	**
Error	11.3	16	0.706		
Total	163	24			

Note: ** indicates significance at $p < 0.01$ (F-critical ($df_1 = 4$, $df_2 = 16$, $\alpha = 0.01$) = 4.77).

Table S38. Comprehensive experimental results for iron-bound humin (HMi-2) alkaline extraction in farmland soil.

Run	Time (h)	Concentration (mol L^{-1})	OM content (g kg^{-1})
1	2	0.05	0.0378
2	2	0.1	0.224
3	2	0.5	0.995
4	2	1.0	1.06
5	2	2.0	1.07
6	5	0.05	0.0459
7	5	0.1	0.272
8	5	0.5	1.17
9	5	1.0	1.14
10	5	2.0	1.12
11	8	0.05	0.0513
12	8	0.1	0.304
13	8	0.5	1.28
14	8	1.0	1.23
15	8	2.0	1.23
16	12	0.05	0.0540
17	12	0.1	0.341
18	12	0.5	1.53
19	12	1.0	1.53
20	12	2.0	1.53
21	24	0.05	0.0553
22	24	0.1	0.332
23	24	0.5	1.46
24	24	1.0	1.43
25	24	2.0	1.44

Table S39. Range analysis for iron-bound humin (HMi-2) alkaline extraction in farmland soil.

Factor	k1	k2	k3	k4	k5	R
Time (h)	0.676	0.748	0.819	0.996	0.942	0.320
Concentration (mol L^{-1})	0.0489	0.295	1.29	1.27	1.28	1.24

Table S40. Analysis of variance (ANOVA) for iron-bound humin (HMi-2) alkaline extraction in farmland soil.

Source	SS	df	MS	F	Significance
Time	0.352	4	0.0880	8.78	**
Concentration	7.51	4	1.88	187	**
Error	0.160	16	0.0100		
Total	8.02	24			

Note: ** indicates significance at $p < 0.01$ (F-critical ($df_1 = 4$, $df_2 = 16$, $\alpha = 0.01$) = 4.77).

Table S41. Comprehensive experimental results for clay-bound humin (HMc-1) acid extraction in forest soil.

Run	Time (h)	Concentration (mol L^{-1})	OM content (g kg^{-1})
1	2	0.05	0.0461
2	2	0.1	0.232
3	2	0.5	0.422
4	2	1.0	0.510
5	2	2.0	0.516
6	5	0.05	0.0560
7	5	0.1	0.282
8	5	0.5	0.512
9	5	1.0	0.637
10	5	2.0	0.625
11	8	0.05	0.0626
12	8	0.1	0.315
13	8	0.5	0.572
14	8	1.0	0.698
15	8	2.0	0.715
16	12	0.05	0.0659
17	12	0.1	0.332
18	12	0.5	0.602
19	12	1.0	0.774
20	12	2.0	0.756
21	24	0.05	0.0673
22	24	0.1	0.337
23	24	0.5	0.593
24	24	1.0	0.753
25	24	2.0	0.751

Table S42. Range analysis for clay-bound humin (HMc-1) acid extraction in forest soil.

Factor	k1	k2	k3	k4	k5	R
Time (h)	0.345	0.422	0.473	0.506	0.500	0.161
Concentration (mol L^{-1})	0.0596	0.300	0.540	0.674	0.673	0.615

Table S43. Analysis of variance (ANOVA) for clay-bound humin (HMc-1) acid extraction in forest soil.

Source	SS	df	MS	F	Significance
Time	0.0895	4	0.0224	13.0	**
Concentration	1.42	4	0.354	206	**
Error	0.0275	16	0.00172		
Total	1.53	24			

Note: ** indicates significance at $p < 0.01$ (F-critical ($df_1 = 4$, $df_2 = 16$, $\alpha = 0.01$) = 4.77).

Table S44. Comprehensive experimental results for clay-bound humin (HMc-1) acid extraction in vegetable soil.

Run	Time (h)	Concentration (mol L^{-1})	OM content (g kg^{-1})
1	2	0.05	0.0805
2	2	0.1	0.526
3	2	0.5	0.816
4	2	1.0	0.896
5	2	2.0	0.905
6	5	0.05	0.0978
7	5	0.1	0.639
8	5	0.5	0.990
9	5	1.0	1.19
10	5	2.0	1.11
11	8	0.05	0.109
12	8	0.1	0.714
13	8	0.5	1.11
14	8	1.0	1.22
15	8	2.0	1.22
16	12	0.05	0.115
17	12	0.1	0.752
18	12	0.5	1.17
19	12	1.0	1.34
20	12	2.0	1.32
21	24	0.05	0.119
22	24	0.1	0.775
23	24	0.5	1.18
24	24	1.0	1.31
25	24	2.0	1.31

Table S45. Range analysis for clay-bound humin (HMc-1) acid extraction in vegetable soil.

Factor	k1	k2	k3	k4	k5	R
Time (h)	0.645	0.806	0.874	0.940	0.940	0.295
Concentration (mol L^{-1})	0.104	0.681	1.05	1.19	1.18	1.09

Table S46. Analysis of variance (ANOVA) for clay-bound humin (HMc-1) acid extraction in vegetable soil.

Source	SS	df	MS	F	Significance
Time	0.302	4	0.0754	15.6	**
Concentration	4.23	4	1.06	220	**
Error	0.0771	16	0.00482		
Total	4.61	24			

Note: ** indicates significance at $p < 0.01$ (F-critical ($df_1 = 4$, $df_2 = 16$, $\alpha = 0.01$) = 4.77).

Table S47. Comprehensive experimental results for clay-bound humin (HMc-1) acid extraction in farmland soil.

Run	Time (h)	Concentration (mol L^{-1})	OM content (g kg^{-1})
1	2	0.05	0.0217
2	2	0.1	0.181
3	2	0.5	0.429
4	2	1.0	0.499
5	2	2.0	0.507
6	5	0.05	0.0264
7	5	0.1	0.219
8	5	0.5	0.521
9	5	1.0	0.593
10	5	2.0	0.574
11	8	0.05	0.0295
12	8	0.1	0.245
13	8	0.5	0.582
14	8	1.0	0.663
15	8	2.0	0.627
16	12	0.05	0.0310
17	12	0.1	0.258
18	12	0.5	0.613
19	12	1.0	0.728
20	12	2.0	0.688
21	24	0.05	0.0320
22	24	0.1	0.265
23	24	0.5	0.623
24	24	1.0	0.720
25	24	2.0	0.665

Table S48. Range analysis for clay-bound humin (HMc-1) acid extraction in farmland soil.

Factor	k1	k2	k3	k4	k5	R
Time (h)	0.327	0.387	0.429	0.464	0.461	0.136
Concentration (mol L^{-1})	0.0281	0.234	0.554	0.641	0.612	0.613

Table S49. Analysis of variance (ANOVA) for clay-bound humin (HMc-1) acid extraction in farmland soil.

Source	SS	df	MS	F	Significance
Time	0.0657	4	0.0164	11.5	**
Concentration	1.46	4	0.364	255	**
Error	0.0229	16	0.00143		
Total	1.55	24			

Note: ** indicates significance at $p < 0.01$ (F-critical ($df_1 = 4$, $df_2 = 16$, $\alpha = 0.01$) = 4.77).

Table S50. Comprehensive experimental results for clay-bound humin (HMc-2) alkaline extraction in forest soil.

Run	Time (h)	Concentration (mol L^{-1})	OM content (g kg^{-1})
1	2	0.05	0.0483
2	2	0.1	0.359
3	2	0.5	0.760
4	2	1.0	0.788
5	2	2.0	0.886
6	5	0.05	0.0587
7	5	0.1	0.436
8	5	0.5	0.961
9	5	1.0	0.951
10	5	2.0	1.03
11	8	0.05	0.0656
12	8	0.1	0.487
13	8	0.5	1.35
14	8	1.0	1.27
15	8	2.0	1.26
16	12	0.05	0.0690
17	12	0.1	0.513
18	12	0.5	1.69
19	12	1.0	1.63
20	12	2.0	1.63
21	24	0.05	0.0705
22	24	0.1	0.524
23	24	0.5	1.66
24	24	1.0	1.62
25	24	2.0	1.61

Table S51. Range analysis for clay-bound humin (HMc-2) alkaline extraction in forest soil.

Factor	k1	k2	k3	k4	k5	R
Time (h)	0.568	0.686	0.885	1.11	1.10	0.539
Concentration (mol L^{-1})	0.0624	0.464	1.29	1.25	1.28	1.22

Table S52. Analysis of variance (ANOVA) for clay-bound humin (HMc-2) alkaline extraction in forest soil.

Source	SS	df	MS	F	Significance
Time	1.16	4	0.291	7.96	**
Concentration	6.52	4	1.63	44.6	**
Error	0.585	16	0.0366		
Total	8.27	24			

Note: ** indicates significance at $p < 0.01$ (F-critical ($df_1 = 4$, $df_2 = 16$, $\alpha = 0.01$) = 4.77).

Table S53. Comprehensive experimental results for clay-bound humin (HMc-2) alkaline extraction in vegetable soil.

Run	Time (h)	Concentration (mol L^{-1})	OM content (g kg^{-1})
1	2	0.05	0.173
2	2	0.1	0.378
3	2	0.5	2.19
4	2	1.0	2.23
5	2	2.0	2.25
6	5	0.05	0.210
7	5	0.1	0.459
8	5	0.5	2.24
9	5	1.0	2.42
10	5	2.0	2.46
11	8	0.05	0.235
12	8	0.1	0.513
13	8	0.5	3.21
14	8	1.0	3.06
15	8	2.0	3.06
16	12	0.05	0.247
17	12	0.1	0.540
18	12	0.5	3.70
19	12	1.0	3.55
20	12	2.0	3.54
21	24	0.05	0.252
22	24	0.1	0.551
23	24	0.5	3.64
24	24	1.0	3.52
25	24	2.0	3.50

Table S54. Range analysis for clay-bound humin (HMc-2) alkaline extraction in vegetable soil.

Factor	k1	k2	k3	k4	k5	R
Time (h)	1.44	1.56	2.02	2.31	2.29	0.870
Concentration (mol L^{-1})	0.223	0.488	3.00	2.96	2.96	2.77

Table S55. Analysis of variance (ANOVA) for clay-bound humin (HMc-2) alkaline extraction in vegetable soil.

Source	SS	df	MS	F	Significance
Time	3.30	4	0.826	7.43	**
Concentration	41.2	4	10.3	92.8	**
Error	1.78	16	0.111		
Total	46.3	24			

Note: ** indicates significance at $p < 0.01$ (F-critical ($df_1 = 4$, $df_2 = 16$, $\alpha = 0.01$) = 4.77).

Table S56. Comprehensive experimental results for clay-bound humin (HMc-2) alkaline extraction in farmland soil.

Run	Time (h)	Concentration (mol L^{-1})	OM content (g kg^{-1})
1	2	0.05	0.0322
2	2	0.1	0.224
3	2	0.5	0.987
4	2	1.0	1.08
5	2	2.0	1.09
6	5	0.05	0.0391
7	5	0.1	0.272
8	5	0.5	1.10
9	5	1.0	1.15
10	5	2.0	1.17
11	8	0.05	0.0437
12	8	0.1	0.304
13	8	0.5	1.23
14	8	1.0	1.27
15	8	2.0	1.28
16	12	0.05	0.0460
17	12	0.1	0.320
18	12	0.5	1.46
19	12	1.0	1.35
20	12	2.0	1.34
21	24	0.05	0.0470
22	24	0.1	0.329
23	24	0.5	1.45
24	24	1.0	1.33
25	24	2.0	1.33

Table S57. Range analysis for clay-bound humin (HMc-2) alkaline extraction in farmland soil.

Factor	k1	k2	k3	k4	k5	R
Time (h)	0.683	0.748	0.825	0.903	0.897	0.219
Concentration (mol L^{-1})	0.0416	0.290	1.25	1.24	1.24	1.20

Table S58. Analysis of variance (ANOVA) for clay-bound humin (HMc-2) alkaline extraction in farmland soil.

Source	SS	df	MS	F	Significance
Time	0.181	4	0.0453	7.29	**
Concentration	7.10	4	1.77	285	**
Error	0.0996	16	0.00622		
Total	7.38	24			

Note: ** indicates significance at $p < 0.01$ (F-critical ($df_1 = 4$, $df_2 = 16$, $\alpha = 0.01$) = 4.77).

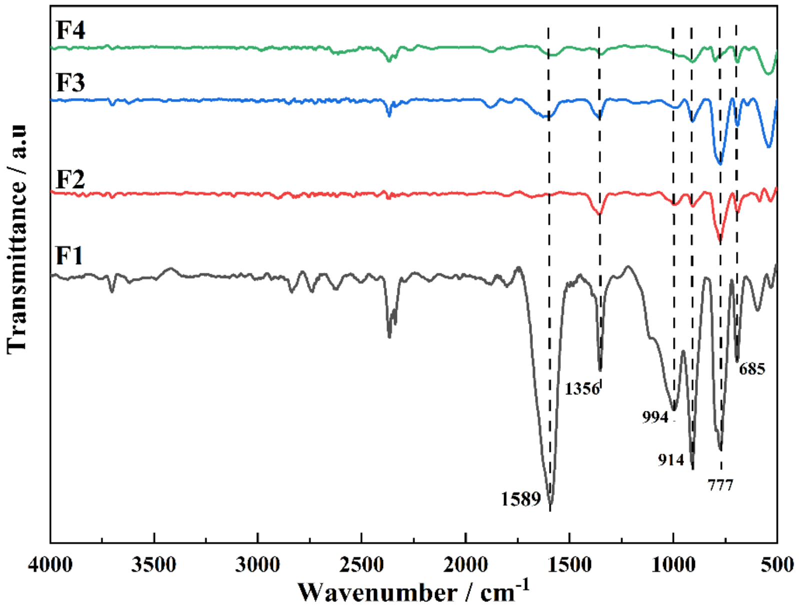

Figure S1. FTIR spectra of the farmland soil after sequential extraction. F1, original soil; F2, after removal of exchangeable fraction (Ex) and humic/fulvic acids (HA/FA); F3, after removal of iron-bound humin (HMi); and F4, after removal of clay-bound humin (HMc).

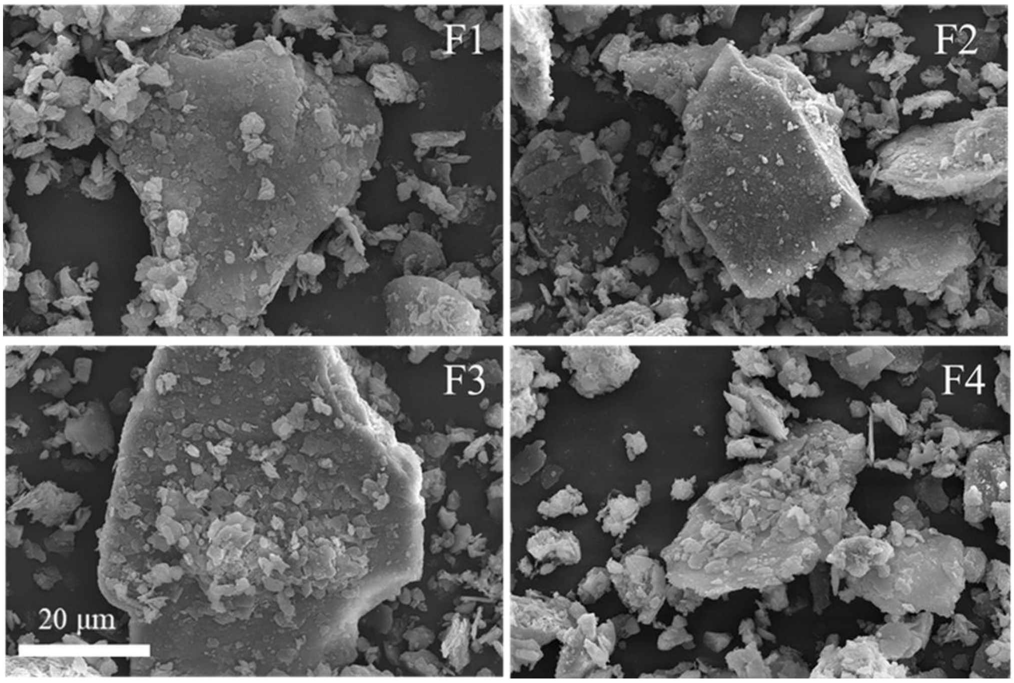

Figure S2. SEM micrographs of the farmland soil after sequential extraction. F1, original soil; F2, after removal of exchangeable fraction (Ex) and humic/fulvic acids (HA/FA); F3, after removal of iron-bound humin (HMi); and F4, after removal of clay-bound humin (HMc).

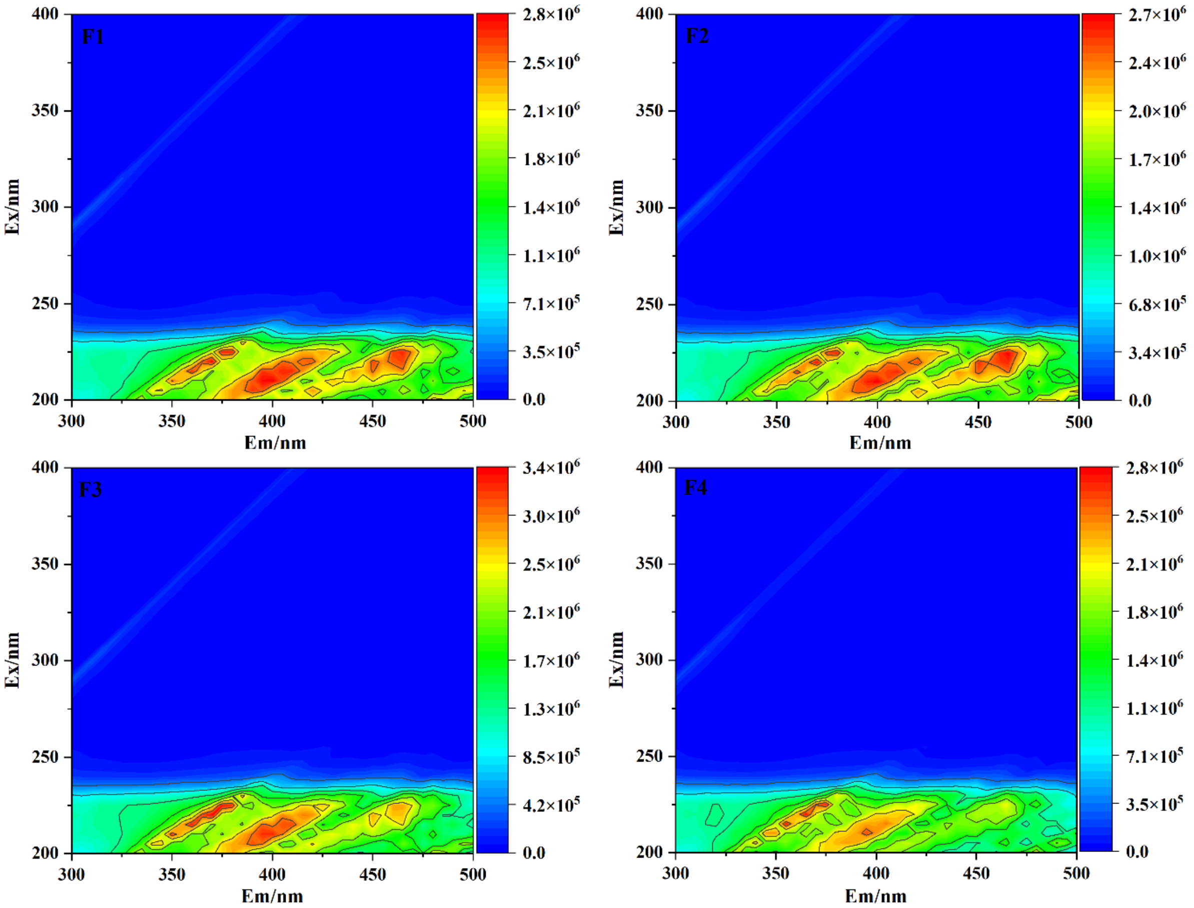

Figure S3. Three-dimensional excitation–emission matrix (EEM) fluorescence spectra of vegetable soil at different extraction stages (F1–F4). F1, original soil; F2, after removal of exchangeable fraction (Ex) and humic/fulvic acids (HA/FA); F3, after removal of iron-bound humin (HMi); and F4, after removal of clay-bound humin (HMc). Each panel uses its own intensity scale.

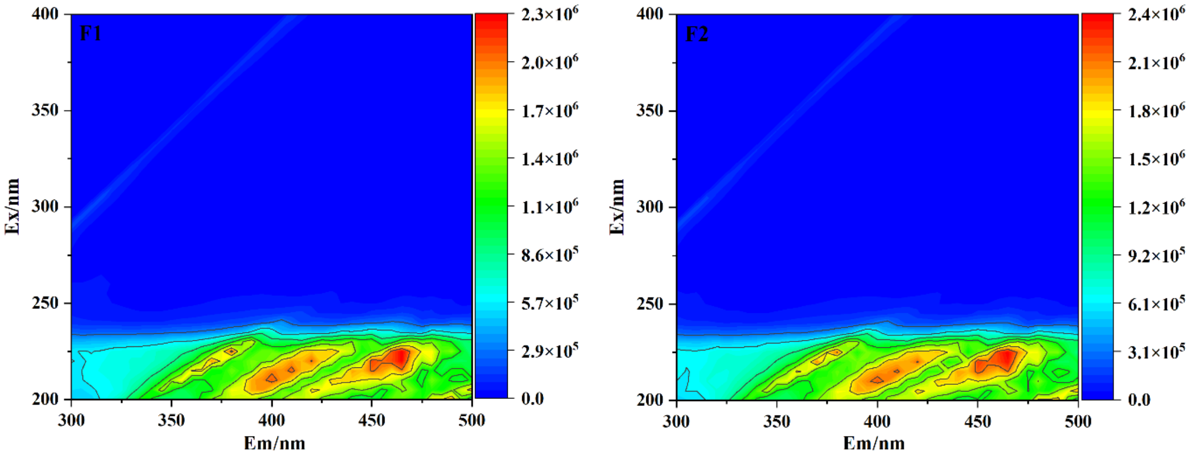

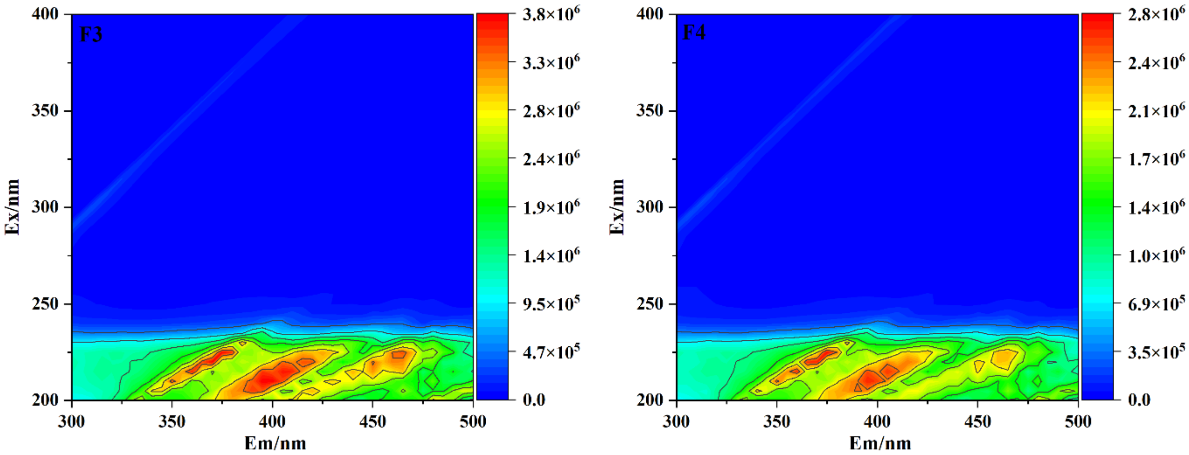

Figure S4. Three-dimensional excitation–emission matrix (EEM) fluorescence spectra of farmland soil at different extraction stages (F1–F4). F1, original soil; F2, after removal of exchangeable fraction (Ex) and humic/fulvic acids (HA/FA); F3, after removal of iron-bound humin (HMi); and F4, after removal of clay-bound humin (HMc). Each panel uses its own intensity scale.
